# The multiplexed single-tier InBios Lyme Detect Multiplex ELISA is more sensitive than standard two-tier tests in the early stages of Lyme disease

**DOI:** 10.1128/jcm.00629-25

**Published:** 2025-10-09

**Authors:** Anna F. Hickman, Allison F. Weber, Elizabeth J. Horn, Peter J. Gwynne

**Affiliations:** 1 Tufts Lyme Disease Initiative, Boston, Massachusetts, USA; 2 Tufts University School of Medicine 12261, Boston, Massachusetts, USA; 3 Lyme Disease Biobank, Portland, Oregon, USA; Mayo Clinic Minnesota, Rochester, Minnesota, USA

**Keywords:** Tickborne disease, diagnosis, serology, Lyme disease

## Abstract

There are nearly 500,000 cases of Lyme disease each year in the United States; 10%–20% of them result in the development of a debilitating chronic disease known as post-treatment Lyme disease. Existing standardized and modified two-tier tests (STT/MTT) suffer from poor detection rates in the first weeks of infection, where the antibody response, the basis of diagnosis, is developing but is not robust enough for detection. During this early window, false negative results are common, which leads to delayed treatment and increases the likelihood of developing severe symptoms. The InBios Lyme Detect Multiplex ELISA is a microarray-based assay designed to capture a set of commonly used diagnostic antibodies specific to *Borrelia burgdorferi* from human serum. The multiplex array captures common diagnostic antibodies, including those to C6, VlsE, and OspC, and has in-line controls. Diagnostic index scores are calculated from the relative abundance of controls and antibodies using a proprietary machine learning algorithm. The assay was evaluated here for reproducibility, accuracy, and performance. It was found to be reproducible using a group of 30 samples run in triplicate. The assay performed well in a blinded panel, correctly identifying all standard two-tier test-positive samples and controls while also detecting 21 of 79 samples that were clinically diagnosed but undetectable by standard Lyme serologic tests. There was one false positive from 66 look-alike disease samples and 146 healthy controls. The InBios assay has the potential to improve diagnostic sensitivity within the early weeks of infection while matching the specificity of current diagnostic tests.

**IMPORTANCE:**

During initial Lyme disease infection, existing diagnostic tests have poor sensitivity, resulting in a high number of false-negative tests. This is due to the lag between infection and a robust immune response capable of being detected by such tests. With a multiplexed array of nine unique antibody targets specific for *Borrelia burgdorferi*, interpreted by a proprietary machine learning algorithm, the InBios Lyme Detect Multiplex ELISA has the potential to increase diagnostic sensitivity within the first few weeks of infection, reducing the number of false-negative tests. Improving diagnostic sensitivity during early infection would reduce the risk of developing severe symptoms, including post-treatment Lyme disease.

## INTRODUCTION

Lyme Disease is the most commonly reported tick-borne disease in the United States and Western Europe. It is caused by spirochetes in the genus *Borrelia:* the majority of the estimated 476,000 (1) Lyme disease cases seen in the United States each year are attributed to *Borrelia burgdorferi*. Cases are most commonly seen in the Northeast, Mid-Atlantic, and Upper-Midwest regions of the United States (2). Early, localized infection typically results in fever, fatigue, and a pathognomonic erythema migrans rash (EM), whereas disseminated infection can spread to the joints, heart, and nervous system to cause more debilitating symptoms later (3). Prompt antibiotic treatment may reduce the risk of the severe side effects seen in later disseminated disease (4), but it may be delayed by insensitive molecular diagnosis in the early stages.

Current guidelines outlined by the Centers for Disease Control and Prevention (CDC) (5) and the Infectious Diseases Society of America (IDSA) (6) recommend a standardized two-tier testing process (STT testing), consisting of an enzyme immunoassay (EIA) or an indirect immunofluorescent antibody assay (IFA) as the first step. If the first tier is positive or equivocal, immunoblots for IgG and IgM antibodies are run to confirm the diagnosis. A recognized alternative approach is MTT testing (modified two-tier testing), in which both of the steps are EIAs, run either consecutively or concurrently. Historically, the most common EIAs used have included the C6 Peptide ELISA (7), whole cell sonicate ELISA (8), and the VlsE/pepC10 ELISA (9).

Existing tests are limited by a well-described “window period” in which the infected patients can remain seronegative in the first weeks after infection, when the antibody response takes time to develop (10). In this early window, antibodies develop against immunogens, including outer surface proteins OspA, OspB, and OspC, decorin binding proteins, and the *erp* proteins (11–13), whereas the most prevalent antibodies are usually directed against VlsE and FlaB epitopes (14). Commercially available tests target different combinations of antigens developed in early infection.

It is within this “window period” that the antibody response develops, where false negative results are common using standard serologic tests. It is not until 3–4 weeks post-infection that the IgG response develops reliably and detectably (15, 16). With STT testing only capable of diagnosing 30%–40% (17) of Lyme disease cases successfully during the first weeks of infection, the current recommendation for clinicians is to avoid using serological tests when an erythema migrans has been present for 2 weeks or less (6). MTT test methods show only slight improvement; when patients have an EM, the tests have 50% sensitivity (18). The low sensitivity of existing serologic tests in the early stages of infection directly impacts patient care as delayed treatment is linked to an increased severity of symptoms and a greater risk of chronic post-treatment symptoms (19).

Therefore, the development of improved testing strategies to shorten the window in which infections are undetectable is a priority. Strategies to improve early detection have included direct detection of antigens (20–22), pathogen DNA (23–25), or inflammatory cytokines via cell-based reporters (26). An alternative host-targeted diagnostic approach is the interferon release assay, in which T cells are cultured from patient blood and stimulated with antigenic peptides to identify Borrelia-specific host responses (27, 28). The multiplexing of numerous antigens, such as in the Viramed ViraChip assay (29), is an approach to maximize the performance of existing ELISA-based technologies. The InBios Lyme Detect Multiplex ELISA is a recently developed system that multiplexes the detection of 11 diagnostic antibodies. The InBios system, consisting of an assay kit containing plates and reagents, a microplate reader, and accompanying analysis software, was evaluated in this study.

## MATERIALS AND METHODS

### Sample collection

The evaluation of the InBios Lyme Detect Multiplex ELISA utilized 45 control samples from a commercial vendor (Pel-Freez) collected in Memphis, Tennessee. Being from a non-endemic collection site, these samples were presumed to be negative. The evaluation also utilized 195 samples from the Lyme Disease Biobank (LDB). Participants were enrolled with signs and symptoms of early Lyme on the East Coast and in the Upper Midwest (30, 31). Endemic control samples were collected from individuals in the same regions without a history of Lyme or other tick-borne infections. Serologic testing, including a first-tier ELISA and IgM and IgG immunoblots, was performed on all samples as previously described (30). The 195 LDB samples included 16 that were positive by STT Testing, 79 samples with EM >5 cm but negative by STT Tests, and 100 endemic controls that were negative on all serologic tests. Samples were blinded for Lyme Detect testing. The evaluation included 15 samples from a commercial vendor. The SeraCare AccuSet Lyme Performance Panel is comprised of single donors with a range of IgM and IgG antibody reactivities across a number of commercial Lyme IgM and IgG tests. Nine of the samples were from the United States, four were unknown, and one was from Germany. Finally, Lyme disease lookalike samples were purchased from commercial vendors. The panel contains many of the lookalike conditions found in the CDC’s Lyme Disease diagnostic evaluation panel (32) and was composed of serum from patients with the following conditions: fibromyalgia (15: Sanguine Biosciences), multiple sclerosis (14: LGC and Sanguine Biosciences), lupus (15: LGC and Sanguine Biosciences), rheumatoid arthritis (10; LGC), and Syphilis (12: Precision for Medicine). Available patient data for all specimens can be found in Tables S1 to S4.

### InBios Lyme Detect Multiplex ELISA plate

**Fig 1:**
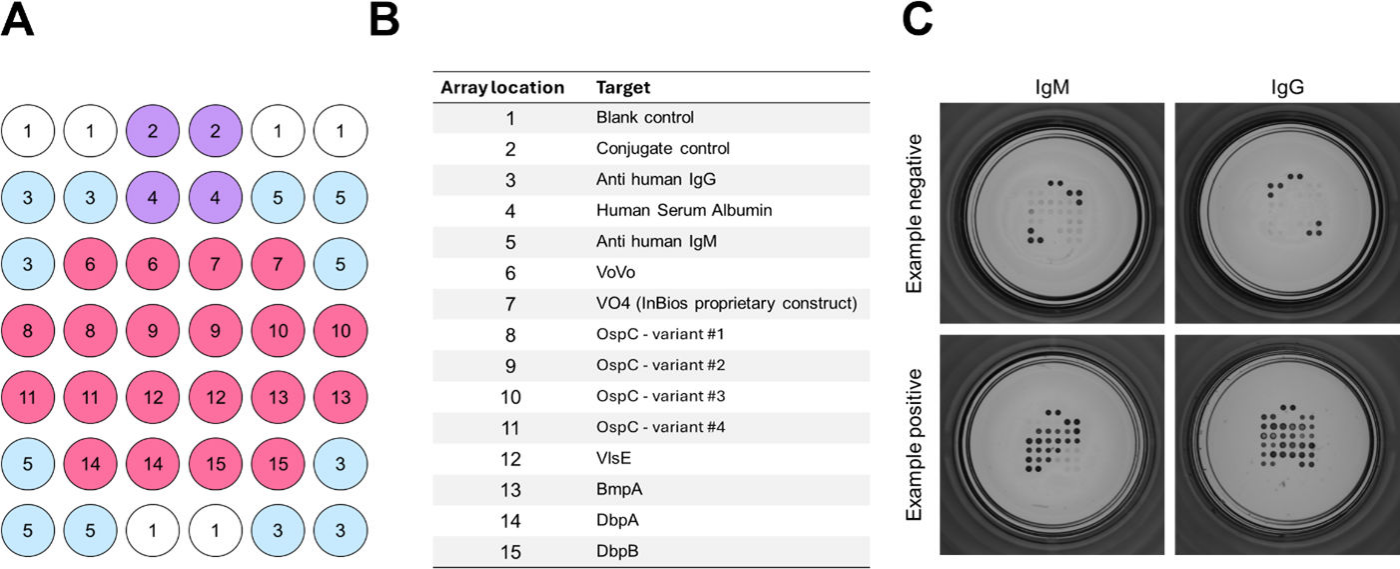
The InBios Lyme Detect Multiplex ELISA is an IgM and IgG microarray-based antibody-capture assay. Each well in the assay is pre-spotted with a microarray of 35 (**A**) stabilized protein spots assaying specific antigens (pink) and internal controls (blue/purple). The internal controls and target antigens (**B**) are printed in triplicate or duplicate. Sample images from InBios QuantoPic software showing experimental positives and negatives for both IgM and IgG are seen in (**C**).

The InBios Lyme Detect Multiplex ELISA is a microarray-based IgM and IgG assay for the capture of antibodies in human serum by a set of target antigens specific for *Borrelia burgdorferi*. The assay utilized a 96-well microtiter plate with 12, 1 × 8 well strips. Each polystyrene well was pre-spotted with a stabilized protein microarray containing purified recombinant antigens (either full-length proteins or shorter epitopes) and internal controls (Fig. 1). Quality control of arrays includes validation of consistent reactivity across a panel of validation specimens.

The internal controls include empty spots (used for orientation confirmation), conjugate control (anti-mouse IgG), human serum albumin (HSA), anti-human IgG antibody, and anti-human IgM antibody. If the assay is performed correctly, human IgG and human IgM antibody reactivity should be observed with IgG and IgM wells, respectively. Every well should show clear reactivity with the conjugate control and minimal to no reactivity with HSA. Spots 6-15 capture specific antibodies present in a suspected active Lyme disease infection. Spot 6 consists of VoVo, a synthetic protein that consists of a combination of VlsE and OspC (33), which are also seen in the array individually. Spots 7-15 are common diagnostic antigens that have been targeted in other microarray or serological assays for Lyme disease detection (10). All samples are normalized to the signal seen in the positive and negative controls, and composite IgM and IgG signal intensities are integrated into an index score, which is the final readout of the assay.

### InBios Lyme Detect Multiplex ELISA protocol

All reagents were equilibrated to room temperature (20–25°C) prior to their use in the assay. Samples were diluted to 1:75 in sample diluent, and 50 µL of both sample and controls was applied to the microtiter plate twice in separate wells (one to be probed for IgM and the other for IgG). The plate was covered and incubated on a shaker set to 500 RPM at 37°C for 1 hour. After the incubation, the plate was washed six times with 300 µL of 1× Wash Buffer per well per wash cycle; 50 µL of 1× IgM and IgG conjugate was applied to the appropriate wells. The plate was covered again before incubation on a shaker set to 500 RPM at 37°C for 30 min. After the incubation, the plate was washed again six times with 300 µL 1× wash buffer per well per wash cycle; 50 µL of precipitating tetramethylbenzidine (TMB) substrate was added to each well before incubation of the plate in the dark for 10 min. The plate was washed twice with 300 µL 1× wash buffer per well per wash cycle and allowed to air dry for 10 min.

The plate was then imaged using the InBios Plate Imager, axiREADER C, model#7N3S2UA#ABA within 60 min of the assay being completed. The raw images were analyzed using the InBios Quantopic v.1.4.3.3 and Multiplex ELISA Analyzer Software v. 1.3.18. The software automatically calculated the signal within each conjugate type and assigned those to each sample or control. The InBios Index Scores were then calculated using a proprietary random forest classifier machine learning model trained on a set of confirmed positive and negative specimens collected from commercial and biobank resources. Three types of model are generated: the total index score incorporates all training data, whereas the IgG and IgM scores are derived from only data from their respective models. Although the specific model type and sample population are considered proprietary, the ML model was generated with these confirmed specimens using standard splits for training and validation of the test results.

### Statistics

Data were graphed using GraphPad Prism 10.4.0, which was also used to perform McNemar’s test, significance testing by Kruskal-Wallis test, followed by Dunn’s post-hoc, Mann-Whitney tests, and receiver-operator characteristic analysis. A support vector machine (SVM) model was trained using the Python library scikit-learn to classify participants as either cases or controls using their InBios Index score. To optimize the model, 5-fold cross-validation was used to tune the gamma and C hyperparameters with a linear kernel.

## RESULTS

### Reproducibility of the assay

Thirty samples were run in triplicate in order to validate the between-run reproducibility of the assay. Fifteen samples (14 reactive and one non-reactive) were previously tested against commercially available Lyme assays by the vendor SeraCare. The panel contained samples with a range of antibody reactivities across several Lyme IgM and IgG test methods. Fourteen of the 15 in the panel were highly positive across the test methods, whereas one (SC15) was negative across all of the testing data provided. The remaining 15 were non-endemic controls and were presumed to be non-reactive.

**Fig 2:**
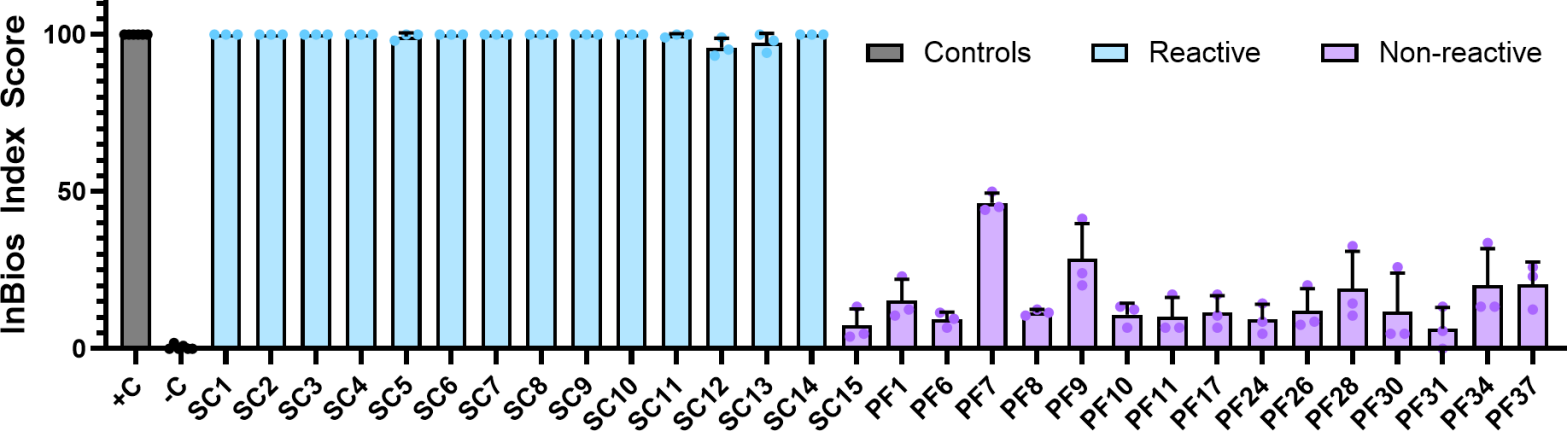
The InBios assay is reproducible across triplicates utilizing well-characterized samples. To determine the between-run reproducibility, the positive and negative controls provided by InBios for each assay, 15 samples from the SeraCare AccuSet Lyme performance panel (SC), as well as 15 non-endemic controls from Pel-Freez (PF) were run in triplicate, whereas the positive and negative controls were run six times. Bars plot the average of the replicates with the error bars showing the standard deviation. All samples were normalized to the positive and negative controls run in parallel.

As shown in Fig. 2, index scores in the positive and negative controls were as expected, with the positive control reading 100 across all three replicates and the negative control ranging between 0.00 and 1.92. The Reactive SeraCare panel had 10 samples with an index score of 100 in all three replicates. The remaining four samples, expected to be highly positive, each averaged greater than 95. The known non-reactive SC15 was grouped with the non-endemic controls. Among the non-reactive group, the highest averaged index score was 46.47, and the lowest was 6.41. There was more variability among this group, although all of the samples were tightly coordinated among the three replicates. These results indicated that the assay was reproducible across runs and validated the experimental design of running the remaining samples in singlicate, as in the intended use case of the assay.

### Comparison of the InBios assay with existing tests

Fifteen samples were supplied with testing data from existing commercial tests (BioMeriuex, MarDx, DiaSorin, and Trinity) detecting Lyme Disease antibodies (Table 1). Index scores were high for all of the first 14 samples and low for SC15. These results were similar in the InBios assay, DiaSorin LIAISON, and the BioMeriuex VIDAS system. The remaining tests had 2–3 samples each that fell below their assay cutoffs. Of the tests that separated IgM and IgG results, the InBios assay was the only test that detected IgG in all of the first 14 samples. IgM detection for the InBios assay matched the other tests, with the IgM in SC11 and 13 going undetected. The InBios Lyme Detect Multiplex ELISA detected more of the Lyme samples than the MarDx EIA Test System and StripBlot Test, and the Trinity Biotech IgG/IgM ELISA, whereas it matches the diagnostic capabilities of the DiaSorin LIAISON system and coupled BioMeriux VIDAS system.

**TABLE 1:** The InBios Lyme Detect Multiplex ELISA matches the diagnostic capabilities of existing tests^*a*^

Sample ID	InBios index score average	InBios IgM index score average	InBios IgG index score average	BioMerieux VIDAS		MarDx EIA test system		MarDx MarBlot strip test		DiaSorin LIAISON Borrelia *burgdorferi* IgM/IgG assay	Trinity biotech Captia Borrelia *burgdorferi* IgG/IgM ELISA
				IgM	IgG	IgM	IgG	IgM	IgG		
**SC1**	**100.00**	**100.00**	**100.00**	**3.00**	**1.20**	**4.20**	0.90	**2/3 P**	3/10 N	**3.60**	**2.00**
**SC2**	**100.00**	**100.00**	**96.51**	**3.10**	0.00	1.10	0.30	1/3 N	2/10 N	**2.10**	0.80
**SC3**	**100.00**	**100.00**	**100.00**	**4.30**	**1.50**	**5.30**	1.00	**2/3 P**	3/10 N	**4.60**	**2.20**
**SC4**	**100.00**	**100.00**	**93.97**	**2.40**	0.10	**4.80**	0.20	**2/3 P**	4/10 N	**2.40**	**2.30**
**SC5**	**99.36**	**100.00**	**99.37**	**4.20**	**0.20**	**2.00**	0.40	**3/3 P**	3/10 N	**6.10**	**1.40**
**SC6**	**100.00**	**100.00**	**98.41**	**3.50**	0.10	**1.30**	0.20	1/3 N	3/10 N	**3.30**	1.00
**SC7**	**100.00**	**100.00**	**100.00**	**4.50**	**0.60**	**4.90**	0.50	**3/3 P**	3/10 N	**3.80**	**2.00**
**SC8**	**100.00**	**82.64**	**100.00**	**0.80**	**2.20**	**2.20**	**1.90**	**3/3 P**	**8/10 P**	**3.90**	**1.80**
**SC9**	**100.00**	**100.00**	**100.00**	**4.10**	**2.40**	**6.70**	0.40	**3/3 P**	3/10 N	**11.00**	**2.40**
**SC10**	**100.00**	**100.00**	**100.00**	**1.30**	**6.70**	0.60	**4.10**	**2/3 P**	**10/10 P**	**>** *12.9,* **>** *12.9*	**2.40**
**SC11**	**99.68**	45.83	**100.00**	0.10	**4.80**	0.10	**1.70**	0/3 N	**10/10 P**	**>** *12.9,12.4*	**1.90**
**SC12**	**95.83**	**94.79**	**90.16**	0.20	**2.40**	0.40	1.00	1/3 P	**6/10 P**	**3.10**	1.00
**SC13**	**97.44**	8.33	**100.00**	0.10	**0.70**	0.40	0.60	0/3 N	**7/10 P**	**4.50**	**1.10**
**SC14**	**100.00**	**99.65**	**100.00**	**0.80**	**1.30**	**2.10**	**1.70**	**2/3 P**	**6/10 P**	**9.70**	**2.20**
**SC15**	7.37	24.31	1.90	0.00	0.00	0.20	0.40	0/3 N	1/10 N	0.00	0.20

^*a*^ 15 samples from the SeraCare AccuSetTM Lyme Performance Panel were run in triplicate on the InBios assay as a direct comparison to the commercially available testing data supplied by the vendor. The InBios assay detects more of the 15 SeraCare (SC) samples than the MarDX EIA and MarBlot Strip Test Systems and the Trinity Biotech Captia ELISA. The InBios assay detects the same number of samples (SC1-14) as the DiaSorin LIAISON and BioMeriuex VIDAS systems. Bolded samples met detection thresholds, unbolded were below detection thresholds, bolded and underlined were equivocal. Bolded and italicized show two replicates reported individually.

### Performance of the InBios assay with blinded samples

**Fig 3:**
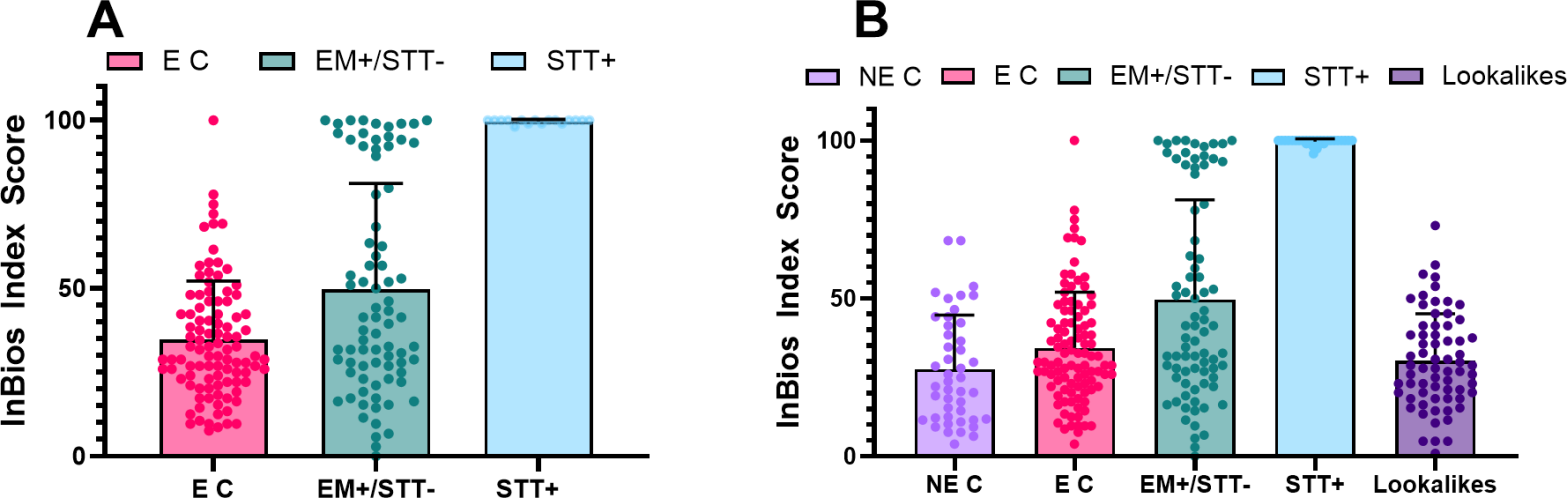
(**A**) The figure shows the blinded LDB panel comprising 100 endemic controls (E C), 79 seronegative Lyme samples (EM+/STT-), and 16 STT positive (STT+) samples run in singlicate. (**B**) The figure collates all analyzed samples with the addition of 45 nonendemic controls (NE C), the SeraCare panel (SC1-14 added to STT+, SC15 added to E C), and 66 look-alike condition samples. Where triplicate data were previously collected, the average is plotted. Endemic controls averaged slightly higher than nonendemic controls, whereas the EM+/STT- group is the most variable and forms two major clusters: the lower group mimics the endemic controls, whereas the upper group mimics the STT+ group. Bars plot the average of the displayed results, and error bars show the standard deviation.

After establishing that the InBios assay matched the diagnostic capabilities of current commercial tests, the performance of the assay on a blinded sample set that mimicked real-world samples was evaluated (Fig. 3A). This sample set consisted of a mix of endemic controls and Lyme patients. The majority of the Lyme patients were clinically diagnosed (by the presence of an EM) but were STT test negative to assess performance in the “window period” where current diagnostics are insensitive. Samples previously run in triplicate (Fig. 2) were averaged, whereas the remaining samples were assayed in singlicate. The blinded LDB panel was comprised of 100 EM negative/STT confirmed negative samples, 79 EM-positive/STT-negative samples, and 16 STT confirmed positive samples.

Figure 3B adds the previous non-endemic controls, SeraCare panel, and 66 samples from look-alike conditions to the data of 3A. Those that were previously run in triplicate (Fig. 2) were averaged, whereas the remaining nonendemic control samples were assayed in singlicate. Kruskal-Wallis test, followed by Dunn’s multiple comparisons test, was used to compare all sample groups. The endemic controls are a population that may have previous exposure to Lyme disease, making it the most representative of a real-world patient population. As expected for a population with possible historic exposure to *B. burgdorferi,* the endemic controls averaged slightly higher at 34.83 compared with the non-endemic controls at 27.59 (*P* > 0.050) and included one sample with an index score of 100.

This sample was negative by all of the other serological tests performed by the Lyme Disease Biobank, and therefore, it appears to be a false positive. The lookalikes group had a similar distribution to the non-endemic and endemic controls, with the highest value being 73.08, and showed no significant difference from them (*P* > 0.050). The STT+ group had high index scores with 22/30 samples at the maximum possible score of 100, and the lowest value was 95.83. This group was significantly higher than the non-endemic and endemic controls and the look-alikes (all *P* < 0.001).

The EM+/STT– group shows two distinct clusters of samples. There is a lower cluster that mirrors the endemic controls with an extended range dragged up by a few outliers and a much tighter group that clusters with index scores of 90–100. The upper group mirrors the spread seen in the STT+ group, which indicates that the InBios assay correctly identifies a portion of the clinically diagnosed but STT test-negative Lyme cases. The EM+/STT− is significantly higher than the non-endemic controls (*P* = 0.001) and lower than the STT+ group (*P* < 0.001), whereas the upper and lower clusters within the EM+/STT− group—separated by a cutoff of 78—are significantly different from each other (*P* < 0.001 by Mann-Whitney test). The lower cluster, notably, is not significantly different from the non-endemic controls (*P* = 0.080), whereas the upper cluster is (*P* < 0.001).

After completing data collection, we analyzed all collected index scores (101 endemic controls, 45 nonendemic controls, 79 EM+/STT−, 30 STT+, and 66 look-alike condition samples) to test the ability of the platform to separate cases from controls. There are numerous approaches to the definition of a diagnostic cutoff, and the interpretation algorithm of the InBios system has not yet been finalized. A number of possible cutoffs derived from the index scores in Fig. 3B are shown in Table 2.

**TABLE 2:** Detection rates in EM+/STT− and STT+ samples using various cutoffs^*a*^

Definition	Cutoff	Probable + ve (%)	Confirmed +ve (%)	Control +ve (%)	Specificity
mean + 2sd	65.46	23 (29.1)	30 (100)	9 (6.16)	95.75
mean + 3sd	82.42	20 (25.3)	30 (100)	1 (0.47)	99.53
**ROC99**	**78.80**	**21** (**26.6**)	**30** (**100**)	**1** (**0.47**)	**99.53**
ROC95	64.90	28 (29.1)	30 (100)	10 (4.72)	95.28
ROC90	54.30	25 (31.6)	30 (100)	19 (8.96)	91.04
SVM	67.32	23 (29.11)	30 (100)	10 (4.69)	95.28

^*a*^ Data from Fig. 3B (confirmed Lyme, and all controls [endemic, nonendemic, and look-alike conditions]) was analyzed. Receiver-operator characteristic (ROC) analysis was performed to identify cutoffs yielding 90%, 95%, or 99% specificity. A cutoff of 78.8 detects 26.6% of the previously undetected EM+/STT− samples with 99.3% specificity (bolded). SVM = Support Vector Machine. SD = standard deviation.

The most common cutoffs for ELISA assays are derived from the mean of the negative control group plus two or three standard deviations. Assuming a normal distribution of index scores, these cutoffs theoretically encompass 95.44% and 99.72% of values expected in the healthy population. The 67.32 cutoff proposed by the SVM model (a supervised machine learning classification algorithm) is the decision boundary that maximizes the margin—the distance between the decision boundary and the point nearest the boundary from each class. The goal of SVM is to increase the likelihood that the model correctly classifies new data points, especially those that fall close to the decision boundary. An alternative approach uses ROC analysis and selecting a cutoff based on a desired specificity or sensitivity milestone. All cutoffs diagnose 100% of confirmed seropositive Lyme samples and additionally detect some of the seronegative Lyme samples. A ROC-derived cutoff of 78.80 (bolded in Table 2) diagnoses 21 (26.6%) of the previously undetectable clinically positive but STT test-negative samples with >99% specificity (a single false positive). This cutoff was used to select a group of STTT-negative Lyme samples for further analysis. However, as some of the non-endemic and endemic controls approached this proposed cutoff, it is important to note that the analysis of more reactive and non-reactive samples has the potential to alter the cutoff value prior to wider use of the device.

### Analysis of the STT-negative/InBios detectable group

There were 79 EM+/STT– samples tested. Based on their positive EM, they were clinically diagnosed with Lyme disease but were negative by the STT testing performed by the Lyme Disease Biobank. Most of the samples were collected in the early weeks of infection, where STT testing is known to perform poorly. The Lyme Disease Biobank provided testing data from the serological tests performed on each sample. Table 3 shows the serological results for the EM+/STT– samples that were positive for at least one of the ELISAs, blots, or surpassed the cutoff of 78.0 for the InBios assay (30/79). When utilizing the cutoff index score of 78.0, the assay had significantly better sensitivity than STT (*P* < 0.001 using McNemar’s test on suspected Lyme cases, *n* = 95). The majority (19/21) of the samples with index scores > 78.0 were also positive by at least one of the other serological assays. All of the samples that were positive on two other serological tests returned InBios Index Scores > 91.0.

**TABLE 3:** Comparison of index scores to previous testing data collected by the Lyme Disease Biobank^*a*^

Sample ID	InBios index score	IgM index score	IgG index score	Whole cell lysate ELISA	C-6 peptide ELISA	VlsE/pepC10 ELISA	Immunoblot IgM	IgM bands	Immunoblot IgG	IgG bands
**LDB14**	**99.04**	64.58	100.00	**POS**	**POS**	NA	NEG	p41	NEG	p66,p41,p39,p28
**LDB17**	**99.04**	98.96	98.10	**POS**	**POS**	NA	NEG	p41	NEG	p39,p41
**LDB39**	**98.08**	100.00	99.05	EQV	**POS**	NA	NEG	p41	NEG	p41
**LDB41**	**95.19**	30.21	99.05	NEG	**NEG**	NA	NEG	p41	NEG	p41,p23
**LDB44**	**89.42**	25.00	98.10	NA	NEG	**POS**	NEG	p41	NEG	p93,p58
**LDB50**	23.08	18.75	31.43	**POS**	NEG	NA	NEG	p41	NEG	p93,p18
**LDB52**	62.50	66.67	62.86	NEG	**POS**	NA	NEG	p41	NEG	p93
**LDB56**	**99.04**	97.92	99.05	NA	**POS**	**POS**	NEG	p41	NEG	None
**LDB60**	28.85	9.38	58.10	NA	NEG	NA	**POS**	p41,p39	NEG	p93,p66
**LDB62**	43.27	56.25	34.29	**POS**	NEG	NA	NEG	p41	NEG	p18
**LDB63**	**92.31**	88.54	94.29	NEG	**POS**	NA	NEG	p41	NEG	None
**LDB65**	14.42	22.92	30.48	**POS**	NEG	NA	NEG	p41	NEG	p93,p41
**LDB66**	**93.27**	75.00	100.00	NEG	**POS**	NA	NEG	p41	NEG	None
**LDB86**	77.88	100.00	30.48	NA	NEG	**POS**	NEG	p23	NEG	None
**LDB89**	**92.31**	79.17	97.14	NA	**POS**	**POS**	NEG	p41	NEG	p41
**LDB91**	**100.00**	84.38	100.00	NA	**POS**	**POS**	NEG	p41	NEG	p41,p39,p23
**LDB98**	27.88	11.46	53.33	NA	**POS**	NEG	NEG	None	NEG	p41,p39
**LDB99**	**99.04**	97.92	98.10	NA	**POS**	**POS**	NEG	p23	NEG	None
**LDB113**	**94.23**	9.38	100.00	NA	**POS**	EQV	NEG	None	NEG	None
**LDB116**	**100.00**	100.00	100.00	NA	**POS**	**POS**	NEG	p23	NEG	p45,p23,p18
**LDB117**	**91.35**	64.58	96.19	NA	**POS**	**POS**	NEG	p41	NEG	p18
**LDB118**	**100.00**	98.96	100.00	NA	**POS**	**POS**	NEG	p41	NEG	p45,p23,p18
**LDB119**	**96.15**	19.79	100.00	NA	**POS**	**POS**	NEG	None	NEG	p66,p45,p39,p18
**LDB132**	53.85	10.42	62.86	NA	NA	**POS**	NEG	p41	NEG	p66,p41,p23
**LDB135**	**100.00**	98.96	100.00	NA	NA	**POS**	NEG	p41	NEG	p93,p41
**LDB136**	**94.23**	100.00	91.43	NA	NA	**POS**	NEG	p23	NEG	p41
**LDB160**	63.46	29.17	64.76	NA	NA	**POS**	NEG	None	NEG	p66,p41
**LDB169**	**96.15**	68.75	99.05	NA	NA	**POS**	NEG	None	NEG	p41,p23
**LDB185**	**79.81**	98.96	62.86	NA	NA	NEG	NEG	None	NEG	None
**LDB188**	**94.23**	88.54	99.05	NA	NA	**POS**	NEG	p41	NEG	p41,p30

^*a*^ 30 samples were analyzed here, all from the LDB blinded panel and clinically positive but STT test negative group (EM+/STT−). The samples included were above the cutoff of 78.00 or were positive for at least one of the serological tests performed by the LDB. The InBios assay detects 21 of the 79 EM+/STT− group, while detecting 100% of the EM+/STT− samples that were positive on two other serological assays and 19 of 21 samples that were positive for at least one other serological test. Bolded samples met detection thresholds, unbolded were below detection thresholds, underlined were equivocal, and tests that were not run for a particular sample were labeled “NA” (not applicable). Bolded samples for the InBios Index Score, InBios IgM Index Score, and InBios IgG Index Score Average met the detection threshold of 78.00 determined through Post-Hoc analysis from data shown in Fig. 3.

**Fig 4:**
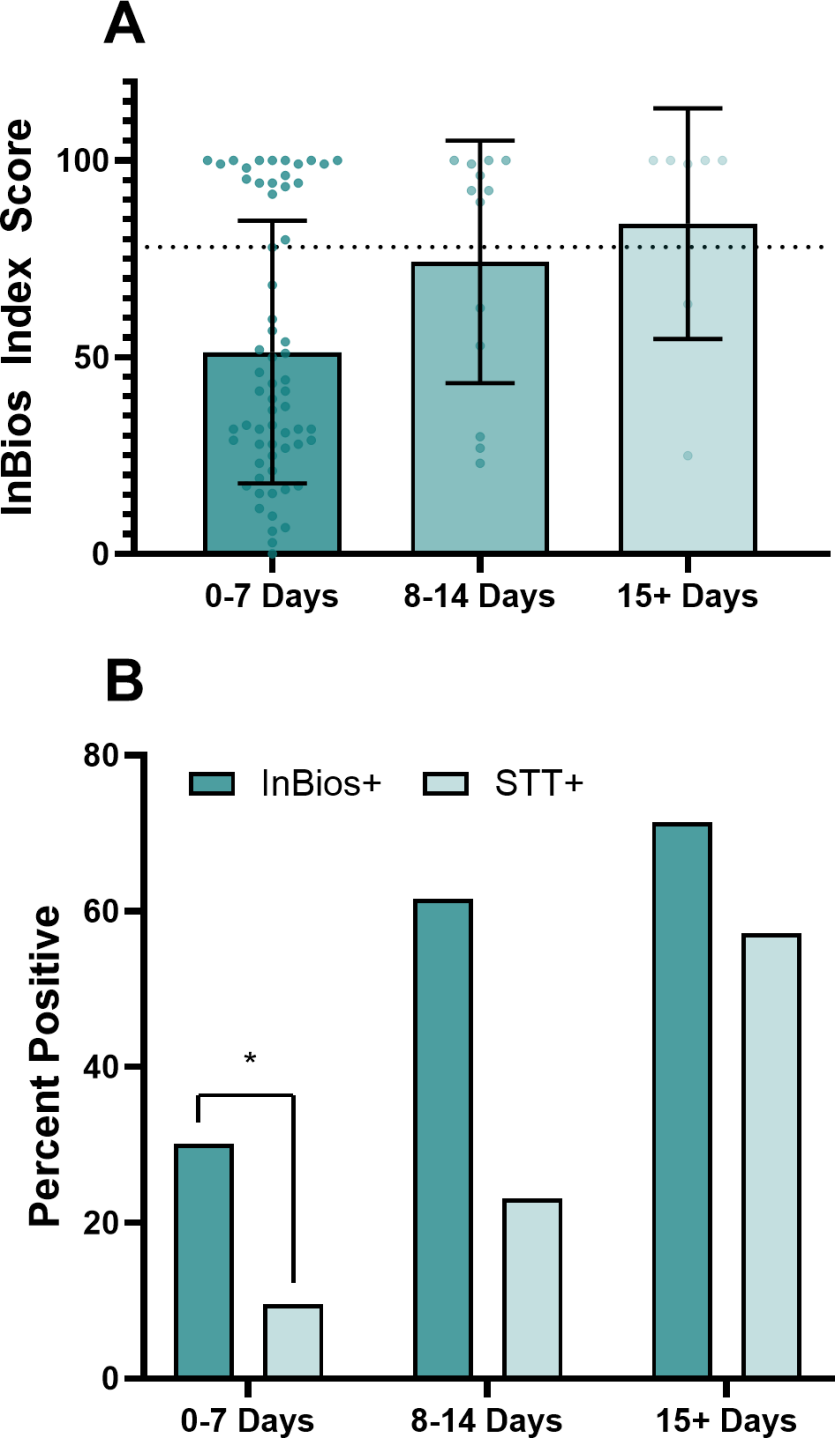
The InBios assay identifies more samples than STT testing at all time points. Eighty-three samples with EM duration data out of 94 patients with reported EM from the LDB Blinded panel (88.30%) were analyzed. (**A**) The figure shows that the average InBios Index Score increases over time for patients who had an EM for 0–7 days, between 8 and 14 days, and 15+ days before sample collection. The dotted line shows the cutoff of 78.00. (**B**) The figure shows the detection rates of the InBios assay (with a cutoff index score of 78) and the STT test. The percentage of InBios+ samples was higher at all time points. There was a significant difference (*P* = <0.05) between the InBios+ and STT+ when the EM lasted a week or less (65 samples) according to McNemar’s test. The study lacked the power to determine significance for the time windows of between 8 and 14 days (11 samples) and above 15 days (7 samples).

Finally, we examined the correlation between the duration of infection and the returned index score. It has been previously established that the longer the length of infection, the higher the likelihood that the sample will be positively identified by STT testing because of the lag between infection and antibody production (15, 16). The precise infection date is usually not known, as patients can rarely identify when they were bitten by a tick. The duration of infection is typically inferred from the first occurrence of the characteristic EM. The association between the duration of EM, InBios index score, and STT testing result is shown in Fig. 4.

Panel A of Fig. 4 shows the index score of all 83 samples with a recorded EM duration over time. As expected, the average Index Score increased over time as the antibody response to the infection developed. Panel B shows the percentage of samples that had an EM for 0–7 days (65), between 8 and 14 days (11), and above 15 days (7) that were above the cutoff of 78.0, compared with samples that were STT+ within those windows. The InBios assay detected more cases than STT testing in all time windows. In the first week of infection, the InBios assay detected 30.16% of all cases, whereas the STT test detected only 9.52%. This was statistically significant by McNemar’s test. With only 11 and 7 samples in the latter two time points, this study lacked the power to determine significance in the time points between 8 and 14 days and 15+ days. As expected (14, 15), the percentage of samples that were STT test positive also increased and was highest among samples that had had an EM for 15 days or longer.

## DISCUSSION

The InBios Lyme Detect system is a straightforward multiplex ELISA, providing single-tier diagnosis of Lyme disease from 4 µL of serum in around 2 hours. The assay was evaluated here for reproducibility, accuracy, and performance in the early weeks of infection, where existing tests suffer poor sensitivity. The test was shown to be reproducible using a group of 30 samples run in triplicate. This reproducibility established the experimental design for the rest of the evaluation: all subsequent samples were run in singlicate, mimicking the intended use of the device.

The assay correctly identified a set of 15 well-characterized Lyme samples. This matched the diagnostic capabilities of both the commercially available DiaSorin LIAISON and the BioMerieux VIDAS systems, demonstrating that the InBios Lyme Detect ELISA can reliably detect Lyme antibodies in a manner equivalent to existing tests. In total, only 30 confirmed positive samples were analyzed here. For an accurate determination of analytical sensitivity in seropositive Lyme disease patients, a larger number of samples will be required.

The clinically diagnosed but STT test-negative samples formed two distinct groups. The larger lower cluster looked similar to the endemic control group and presumably contained patients without a mature antibody response to infection. The smaller upper cluster grouped very similarly to the STT test positive group and represents patients detected by the InBios assay who were not detected by standardized tests. A cutoff of 78 detected 21/79 clinically positive but STT test negative samples, with 100% (30/30) of the STT positive samples detected, and a single false positive out of 146 controls and 66 lookalike condition samples (99.3% specificity). Further analysis of larger sample sets could help both refine the diagnostic cutoff and train the machine learning algorithm used to classify samples.

The multiplexed approach allows the InBios assay to detect more samples than any of the first-tier single antigen ELISAs previously tested. Thirty of the 79 clinically positive but STT test-negative group were positive for one or more serological tests or surpassed the cutoff of 78.00. The majority (19 of the 21) of samples that were over the index score cutoff of 78.00 were also serologically positive by at least one of the Whole Cell Lysate, C6 Peptide, or VlsE/pepC10 ELISAs. The InBios Assay detected half of the EM-positive but STT test-negative samples (9/18) that were positive for only one of the other serological tests. It detected 100% (10/10) of samples that were STT test negative but had positive results on two first-tier serological tests. Thus, in a single-tier test, the InBios assay is able to detect samples that were detected by first-tier serology but negative in the second tier of the STT algorithm. There was no compromise in specificity, which was 99.3% in this study, and no demonstration of cross-reactivity among the lookalike conditions tested.

The assay evaluated here is essentially an ELISA, making it susceptible to typical limitations of immunoassays such as interference from other components of patient sera, such as lipemia and hemolysis (34)—these potential analytical interferences were not tested here. Similarly, we did not test potential clinical confounders such as other tick-borne pathogens. Wider analytical and clinical specificity testing will be needed before adoption of the platform. Another traditional limitation of immunoassays is in the definition of a diagnostic cutoff value, but the machine learning component of the diagnostic algorithm may help to remove some of the subjectivity from this critical decision. However, the details of the algorithm’s training and the exact composition of the antigen array are proprietary and were not shared with the authors; thus, the data presented are limited to an evaluation of the provided materials. A final limitation here is common to all efforts to improve Lyme disease diagnosis. In the absence of a gold-standard test, we are reliant on clinical signs for the selection of patient samples. The seronegative Lyme samples (EM+/STT−) analyzed here were included based on the presence of a clinician-diagnosed erythema migrans rash of >5 cm. This clinical measure is an imperfect diagnostic tool (35), and patients in the EM+/STT− group may have been mischaracterized based on skin rash alone. In this case, sensitivity may be higher than reported here.

We further examined the performance advantage of the InBios test by comparing index scores to STT testing results at intervals of 1 week post-infection. At all time points, measured by the duration of erythema migrans, the InBios assay identifies more samples (using 78.00 as the cutoff) than STT testing. The system under evaluation combines a new multiplexed sample array with a proprietary classification algorithm. Whether the improved clinical sensitivity is a result of improved analytical performance or due to the different diagnostic criteria is unknown.

Nevertheless, the InBios Lyme Detect Multiplex ELISA is a simple, reproducible, effective test and outperforms STT testing in the early stages of disease. Although the test is currently for research use only, it has the potential to improve diagnostic sensitivity within the 30-day detection window.

## SUPPLEMENTAL MATERIAL

Table S1 jcm.00629-25-s0001.docx Demographic data for 15 unblinded SeraCare AccuSet Lyme Performance Panel samples (SC) representing a range of IgM and IgG antibody reactivities to current commercial Lyme disease diagnostic tests.

Table S2 jcm.00629-25-s0002.docx Demographic data for 45 unblinded non-endemic controls (NE C) samples from commercial vendor Pel-Freez (PF).

Table S3 jcm.00629-25-s0003.docx Demographic data for blinded panel from the Lyme Disease Biobank (LDB) comprised of 100 endemic controls (E C), 79 clinically positive but standard two-tier test negative (EM+/STT-), and 16 standard two-tier test positive (STT+) samples.

Table S4 jcm.00629-25-s0004.docx Demographic data for 66 unblinded lookalike condition samples including 15 lupus (LGC and Sanguine Biosciences), 14 multiple sclerosis (LGC and Sanguine Biosciences), 15 fibromyalgia (Sanguine Biosciences), 10 rheumatoid arthritis (LGC), and 12 syphilis (Precision for Medicine) samples.

ASM does not own the copyrights to Supplemental Material that may be linked to, or accessed through, an article. The authors have granted ASM a non-exclusive, world-wide license to publish the Supplemental Material files. Please contact the corresponding author directly for reuse.
