# Supplementary material for: The multiplexed single-tier InBios Lyme Detect Multiplex ELISA is more sensitive than standard two-tier tests in the early stages of Lyme disease
Source: J Clin Microbiol. 2025 Oct 9;63(11):e00629-25. doi: 10.1128/jcm.00629-25 (PMC12607815; doi:10.1128/jcm.00629-25)
Supplement: Table S1 — Demographic data for 15 unblinded SeraCare AccuSet Lyme Performance Panel samples (SC) representing a range of IgM and IgG antibody reactivities to current commercial Lyme disease diagnostic tests. [file jcm.00629-25-s0001.docx]

|  | | | | | | | Does patient report or recall tick bite? | Size of Rash | | Rash characteristics | | | | | | | Days you had rash | Antibiotic Duration (prophylactic) | Name of antibiotic (prophylactic) | Past history of Lyme disease |
| --- | --- | --- | --- | --- | --- | --- | --- | --- | --- | --- | --- | --- | --- | --- | --- | --- | --- | --- | --- | --- |
| Study Label | Classification | Season | Site | Age | Gender | Race | Response/ days since tick bite | Length (cm) | Width (cm) | History of Expansion | Single EM | Multiple EM | Classic "Bulls-Eye" | Homogenous Erythema | Rash prior to today: | Location(s) (please describe) | Open-Ended Response | Duration (days) | Response | Response |
| SC1 | Reactive | 7/25/2018 | United States | NA | NA | NA | NA | NA | NA | NA | NA | NA | NA | NA | NA | NA | NA | NA | NA | NA |
| SC2 | Reactive | 8/12/2018 | United States | NA | NA | NA | NA | NA | NA | NA | NA | NA | NA | NA | NA | NA | NA | NA | NA | NA |
| SC3 | Reactive | 7/17/2018 | United States | NA | NA | NA | NA | NA | NA | NA | NA | NA | NA | NA | NA | NA | NA | NA | NA | NA |
| SC4 | Reactive | 8/23/2018 | United States | NA | NA | NA | NA | NA | NA | NA | NA | NA | NA | NA | NA | NA | NA | NA | NA | NA |
| SC5 | Reactive | 7/17/2017 | United States | NA | NA | NA | NA | NA | NA | NA | NA | NA | NA | NA | NA | NA | NA | NA | NA | NA |
| SC6 | Reactive | 6/15/2018 | N/A | NA | NA | NA | NA | NA | NA | NA | NA | NA | NA | NA | NA | NA | NA | NA | NA | NA |
| SC7 | Reactive | 8/18/2017 | N/A | NA | NA | NA | NA | NA | NA | NA | NA | NA | NA | NA | NA | NA | NA | NA | NA | NA |
| SC8 | Reactive | 5/21/2018 | United States | NA | NA | NA | NA | NA | NA | NA | NA | NA | NA | NA | NA | NA | NA | NA | NA | NA |
| SC9 | Reactive | 7/27/2017 | N/A | NA | NA | NA | NA | NA | NA | NA | NA | NA | NA | NA | NA | NA | NA | NA | NA | NA |
| SC10 | Reactive | 9/15/2018 | United States | NA | NA | NA | NA | NA | NA | NA | NA | NA | NA | NA | NA | NA | NA | NA | NA | NA |
| SC11 | Reactive | 5/23/2018 | United States | NA | NA | NA | NA | NA | NA | NA | NA | NA | NA | NA | NA | NA | NA | NA | NA | NA |
| SC12 | Reactive | 12/15/2010 | Germany | NA | NA | NA | NA | NA | NA | NA | NA | NA | NA | NA | NA | NA | NA | NA | NA | NA |
| SC13 | Reactive | 11/26/2008 | N/A | NA | NA | NA | NA | NA | NA | NA | NA | NA | NA | NA | NA | NA | NA | NA | NA | NA |
| SC14 | Reactive | 10/28/2008 | N/A | NA | NA | NA | NA | NA | NA | NA | NA | NA | NA | NA | NA | NA | NA | NA | NA | NA |
| SC15 | Non-reactive | 1/26/2009 | United States | NA | NA | NA | NA | NA | NA | NA | NA | NA | NA | NA | NA | NA | NA | NA | NA | NA |

Supplemental Table 1. Demographic data for 15 unblinded SeraCare AccuSet Lyme Performance Panel Samples (SC) representing a range of IgM and IgG antibody reactivities to current commercial Lyme Disease diagnostic tests.
