# Supplementary material for: The multiplexed single-tier InBios Lyme Detect Multiplex ELISA is more sensitive than standard two-tier tests in the early stages of Lyme disease
Source: J Clin Microbiol. 2025 Oct 9;63(11):e00629-25. doi: 10.1128/jcm.00629-25 (PMC12607815; doi:10.1128/jcm.00629-25)
Supplement: Table S2 — Demographic data for 45 unblinded non-endemic controls (NE C) samples from commercial vendor Pel-Freez (PF). [file jcm.00629-25-s0002.docx]

|  | | | | | | | Does patient report or recall tick bite? | Size of Rash | | Rash characteristics | | | | | | | Days you had rash | Antibiotic Duration (prophylactic) | Name of antibiotic (prophylactic) | Past history of Lyme disease |
| --- | --- | --- | --- | --- | --- | --- | --- | --- | --- | --- | --- | --- | --- | --- | --- | --- | --- | --- | --- | --- |
| Study Label | Classification | Season | Site | Age | Gender | Race | Response/ days since tick bite | Length (cm) | Width (cm) | History of Expansion | Single EM | Multiple EM | Classic "Bulls-Eye" | Homogenous Erythema | Rash prior to today: | Location(s) (please describe) | Open-Ended Response | Duration (days) | Response | Response |
| PF1 | Non-Endemic Control | NA | TN | 59 | Male | Black | NA | NA | NA | NA | NA | NA | NA | NA | NA | NA | NA | NA | NA | NA |
| PF2 | Non-Endemic Control | NA | TN | 26 | Male | Black | NA | NA | NA | NA | NA | NA | NA | NA | NA | NA | NA | NA | NA | NA |
| PF3 | Non-Endemic Control | NA | TN | 46 | Female | Black | NA | NA | NA | NA | NA | NA | NA | NA | NA | NA | NA | NA | NA | NA |
| PF4 | Non-Endemic Control | NA | TN | 31 | Male | Black | NA | NA | NA | NA | NA | NA | NA | NA | NA | NA | NA | NA | NA | NA |
| PF5 | Non-Endemic Control | NA | TN | 32 | Female | Latino | NA | NA | NA | NA | NA | NA | NA | NA | NA | NA | NA | NA | NA | NA |
| PF6 | Non-Endemic Control | NA | TN | 42 | Female | Latino | NA | NA | NA | NA | NA | NA | NA | NA | NA | NA | NA | NA | NA | NA |
| PF7 | Non-Endemic Control | NA | TN | 40 | Female | Black | NA | NA | NA | NA | NA | NA | NA | NA | NA | NA | NA | NA | NA | NA |
| PF8 | Non-Endemic Control | NA | TN | 24 | Male | Black | NA | NA | NA | NA | NA | NA | NA | NA | NA | NA | NA | NA | NA | NA |
| PF9 | Non-Endemic Control | NA | TN | 33 | Male | Black | NA | NA | NA | NA | NA | NA | NA | NA | NA | NA | NA | NA | NA | NA |
| PF10 | Non-Endemic Control | NA | TN | 39 | Male | Black | NA | NA | NA | NA | NA | NA | NA | NA | NA | NA | NA | NA | NA | NA |
| PF11 | Non-Endemic Control | NA | TN | 59 | Male | Black | NA | NA | NA | NA | NA | NA | NA | NA | NA | NA | NA | NA | NA | NA |
| PF12 | Non-Endemic Control | NA | TN | 37 | Female | Black | NA | NA | NA | NA | NA | NA | NA | NA | NA | NA | NA | NA | NA | NA |
| PF13 | Non-Endemic Control | NA | TN | 30 | Female | Black | NA | NA | NA | NA | NA | NA | NA | NA | NA | NA | NA | NA | NA | NA |
| PF14 | Non-Endemic Control | NA | TN | 32 | Female | Black | NA | NA | NA | NA | NA | NA | NA | NA | NA | NA | NA | NA | NA | NA |
| PF15 | Non-Endemic Control | NA | TN | 28 | Female | Black | NA | NA | NA | NA | NA | NA | NA | NA | NA | NA | NA | NA | NA | NA |
| PF16 | Non-Endemic Control | NA | TN | 39 | Female | Black | NA | NA | NA | NA | NA | NA | NA | NA | NA | NA | NA | NA | NA | NA |
| PF17 | Non-Endemic Control | NA | TN | 51 | Male | Black | NA | NA | NA | NA | NA | NA | NA | NA | NA | NA | NA | NA | NA | NA |
| PF18 | Non-Endemic Control | NA | TN | 39 | Male | Black | NA | NA | NA | NA | NA | NA | NA | NA | NA | NA | NA | NA | NA | NA |
| PF19 | Non-Endemic Control | NA | TN | 34 | Female | Black | NA | NA | NA | NA | NA | NA | NA | NA | NA | NA | NA | NA | NA | NA |
| PF20 | Non-Endemic Control | NA | TN | 69 | Male | Black | NA | NA | NA | NA | NA | NA | NA | NA | NA | NA | NA | NA | NA | NA |
| PF21 | Non-Endemic Control | NA | TN | 49 | Female | Black | NA | NA | NA | NA | NA | NA | NA | NA | NA | NA | NA | NA | NA | NA |
| PF22 | Non-Endemic Control | NA | TN | 34 | Male | Black | NA | NA | NA | NA | NA | NA | NA | NA | NA | NA | NA | NA | NA | NA |
| PF23 | Non-Endemic Control | NA | TN | 47 | Female | Black | NA | NA | NA | NA | NA | NA | NA | NA | NA | NA | NA | NA | NA | NA |
| PF24 | Non-Endemic Control | NA | TN | 65 | Male | Black | NA | NA | NA | NA | NA | NA | NA | NA | NA | NA | NA | NA | NA | NA |
| PF25 | Non-Endemic Control | NA | TN | 43 | Male | Black | NA | NA | NA | NA | NA | NA | NA | NA | NA | NA | NA | NA | NA | NA |
| PF26 | Non-Endemic Control | NA | TN | 50 | Male | Black | NA | NA | NA | NA | NA | NA | NA | NA | NA | NA | NA | NA | NA | NA |
| PF27 | Non-Endemic Control | NA | TN | 24 | Male | Black | NA | NA | NA | NA | NA | NA | NA | NA | NA | NA | NA | NA | NA | NA |
| PF28 | Non-Endemic Control | NA | TN | 61 | Male | Black | NA | NA | NA | NA | NA | NA | NA | NA | NA | NA | NA | NA | NA | NA |
| PF29 | Non-Endemic Control | NA | TN | 30 | Female | Black | NA | NA | NA | NA | NA | NA | NA | NA | NA | NA | NA | NA | NA | NA |
| PF30 | Non-Endemic Control | NA | TN | 26 | Male | Black | NA | NA | NA | NA | NA | NA | NA | NA | NA | NA | NA | NA | NA | NA |
| PF31 | Non-Endemic Control | NA | TN | 47 | Male | Black | NA | NA | NA | NA | NA | NA | NA | NA | NA | NA | NA | NA | NA | NA |
| PF32 | Non-Endemic Control | NA | TN | 26 | Female | Black | NA | NA | NA | NA | NA | NA | NA | NA | NA | NA | NA | NA | NA | NA |
| PF33 | Non-Endemic Control | NA | TN | 34 | Male | Black | NA | NA | NA | NA | NA | NA | NA | NA | NA | NA | NA | NA | NA | NA |
| PF34 | Non-Endemic Control | NA | TN | 62 | Male | Black | NA | NA | NA | NA | NA | NA | NA | NA | NA | NA | NA | NA | NA | NA |
| PF35 | Non-Endemic Control | NA | TN | 40 | Male | Black | NA | NA | NA | NA | NA | NA | NA | NA | NA | NA | NA | NA | NA | NA |
| PF36 | Non-Endemic Control | NA | TN | 37 | Male | Black | NA | NA | NA | NA | NA | NA | NA | NA | NA | NA | NA | NA | NA | NA |
| PF37 | Non-Endemic Control | NA | TN | 38 | Male | Black | NA | NA | NA | NA | NA | NA | NA | NA | NA | NA | NA | NA | NA | NA |
| PF38 | Non-Endemic Control | NA | TN | 56 | Male | Black | NA | NA | NA | NA | NA | NA | NA | NA | NA | NA | NA | NA | NA | NA |
| PF39 | Non-Endemic Control | NA | TN | 61 | Male | Black | NA | NA | NA | NA | NA | NA | NA | NA | NA | NA | NA | NA | NA | NA |
| PF40 | Non-Endemic Control | NA | TN | 54 | Male | Black | NA | NA | NA | NA | NA | NA | NA | NA | NA | NA | NA | NA | NA | NA |
| PF41 | Non-Endemic Control | NA | TN | 48 | Male | Black | NA | NA | NA | NA | NA | NA | NA | NA | NA | NA | NA | NA | NA | NA |
| PF42 | Non-Endemic Control | NA | TN | 38 | Male | Black | NA | NA | NA | NA | NA | NA | NA | NA | NA | NA | NA | NA | NA | NA |
| PF43 | Non-Endemic Control | NA | TN | 25 | Female | Black | NA | NA | NA | NA | NA | NA | NA | NA | NA | NA | NA | NA | NA | NA |
| PF44 | Non-Endemic Control | NA | TN | 68 | Female | Black | NA | NA | NA | NA | NA | NA | NA | NA | NA | NA | NA | NA | NA | NA |
| PF45 | Non-Endemic Control | NA | TN | 18 | Male | Black | NA | NA | NA | NA | NA | NA | NA | NA | NA | NA | NA | NA | NA | NA |

Supplemental Table 2. Demographic data for 45 unblinded non-endemic controls (NE C) samples from commercial vendor Pel-Freez (PF).
