# Supplementary material for: The multiplexed single-tier InBios Lyme Detect Multiplex ELISA is more sensitive than standard two-tier tests in the early stages of Lyme disease
Source: J Clin Microbiol. 2025 Oct 9;63(11):e00629-25. doi: 10.1128/jcm.00629-25 (PMC12607815; doi:10.1128/jcm.00629-25)
Supplement: Table S3 — Demographic data for blinded panel from the Lyme Disease Biobank (LDB) comprised of 100 endemic controls (E C), 79 clinically positive but standard two-tier test negative (EM+/STT-), and 16 standard two-tier test positive (STT+) samples. [file jcm.00629-25-s0003.docx]

|  | | | | | | | Does patient report or recall tick bite? | Size of Rash | | Rash characteristics | | | | | | | Days you had rash | Antibiotic Duration (prophylactic) | Name of antibiotic (prophylactic) | Past history of Lyme disease |
| --- | --- | --- | --- | --- | --- | --- | --- | --- | --- | --- | --- | --- | --- | --- | --- | --- | --- | --- | --- | --- |
| Study Label | *Classification* | Season | Site | Age | Gender | Race | Response/ days since tick bite | Length (cm) | Width (cm) | History of Expansion | Single EM | Multiple EM | Classic "Bulls-Eye" | Homogenous Erythema | Rash prior to today: | Location(s) (please describe) | Open-Ended Response | Duration (days) | Response | Response |
| LDB1 | Endemic Control | 2014 | EH | 27 | Male | Hispanic or Latino | NA | NA | NA | NA | NA | NA | NA | NA | NA | NA | NA | NA | NA | No |
| LDB2 | Endemic Control | 2014 | EH | 49 | Male | White | NA | NA | NA | NA | NA | NA | NA | NA | NA | NA | NA | NA | NA | No |
| LDB3 | Endemic Control | 2014 | EH | 38 | Female | White | NA | NA | NA | NA | NA | NA | NA | NA | NA | NA | NA | NA | NA | No |
| LDB4 | Endemic Control | 2014 | EH | 22 | Male | Hispanic or Latino | NA | NA | NA | NA | NA | NA | NA | NA | NA | NA | NA | NA | NA | No |
| LDB5 | Endemic Control | 2014 | EH | 21 | Female | Hispanic or Latino | NA | NA | NA | NA | NA | NA | NA | NA | NA | NA | NA | NA | NA | No |
| LDB6 | Endemic Control | 2015 | EH | 29 | Female | White | NA | NA | NA | NA | NA | NA | NA | NA | NA | NA | NA | NA | NA | No |
| LDB7 | Endemic Control | 2015 | EH | 55 | Female | White | NA | NA | NA | NA | NA | NA | NA | NA | NA | NA | NA | NA | NA | No |
| LDB8 | Endemic Control | 2015 | EH | 47 | Male | White | NA | NA | NA | NA | NA | NA | NA | NA | NA | NA | NA | NA | NA | No |
| LDB9 | Endemic Control | 2015 | EH | 53 | Female | White | NA | NA | NA | NA | NA | NA | NA | NA | NA | NA | NA | NA | NA | No |
| LDB10 | Endemic Control | 2015 | EH | 65 | Female | White | NA | NA | NA | NA | NA | NA | NA | NA | NA | NA | NA | NA | NA | No |
| LDB11 | Endemic Control | 2015 | EH | 59 | Male | White | NA | NA | NA | NA | NA | NA | NA | NA | NA | NA | NA | NA | NA | No |
| LDB12 | Endemic Control | 2015 | MV | 69 | Male | White | NA | NA | NA | NA | NA | NA | NA | NA | NA | NA | NA | NA | NA | No |
| LDB13 | Endemic Control | 2015 | EH | 77 | Female | White | NA | NA | NA | NA | NA | NA | NA | NA | NA | NA | NA | NA | NA | No |
| LDB14 | EM+/STT- | 2015 | MV | 47 | Female | White | Yes, 14 | 15 | 23 | Yes | Yes | No | Yes | No | Yes | ARM PIT | NA | NA | NA | Yes |
| LDB15 | Endemic Control | 2016 | EH | 68 | Male | White | NA | NA | NA | NA | NA | NA | NA | NA | NA | NA | NA | NA | NA | No |
| LDB16 | Endemic Control | 2016 | EH | 68 | Female | White | NA | NA | NA | NA | NA | NA | NA | NA | NA | NA | NA | NA | NA | No |
| LDB17 | EM+/STT- | 2016 | EH | 53 | Female | White | Yes, 20 | 14 | 12 | Yes | No | Yes | No | No | Yes | Right thigh | NA | NA | NA | No |
| LDB18 | EM+/STT- | 2016 | WI | 67 | Female | White | No | 6 | 6 | No | Yes | No | No | No | Yes | Left lower leg | NA | 1 | Doxycycline | No |
| LDB19 | Endemic Control | 2016 | EH | 72 | Female | White | NA | NA | NA | NA | NA | NA | NA | NA | NA | NA | NA | NA | NA | No |
| LDB20 | EM+/STT- | 2016 | WI | 51 | Male | White | Yes, 4 | 8 | 5 | Yes | Yes | No | Yes | No | Yes | Left chest wall | NA | NA | NA | No |
| LDB21 | Endemic Control | 2016 | EH | 62 | Male | Hispanic or Latino | NA | NA | NA | NA | NA | NA | NA | NA | NA | NA | NA | NA | NA | No |
| LDB22 | EM+/STT- | 2016 | WI | 63 | Female | White | Yes, 1 | 3 | 7 | No | Yes | No | No | Yes | No | Left side of right knee | NA | NA | NA | No |
| LDB23 | Endemic Control | 2016 | EH | 28 | Male | Hispanic or Latino | NA | NA | NA | NA | NA | NA | NA | NA | NA | NA | NA | NA | NA | No |
| LDB24 | Endemic Control | 2016 | EH | 77 | Male | White | NA | NA | NA | NA | NA | NA | NA | NA | NA | NA | NA | NA | NA | No |
| LDB25 | Endemic Control | 2016 | EH | 59 | Male | White | NA | NA | NA | NA | NA | NA | NA | NA | NA | NA | NA | NA | NA | No |
| LDB26 | Endemic Control | 2016 | EH | 40 | Female | Hispanic or Latino | NA | NA | NA | NA | NA | NA | NA | NA | NA | NA | NA | NA | NA | No |
| LDB27 | Endemic Control | 2016 | EH | 45 | Female | White | NA | NA | NA | NA | NA | NA | NA | NA | NA | NA | NA | NA | NA | No |
| LDB28 | Endemic Control | 2016 | EH | 35 | Male | Hispanic or Latino | NA | NA | NA | NA | NA | NA | NA | NA | NA | NA | NA | NA | NA | No |
| LDB29 | STT+ | 2016 | WI | 47 | Male | White | No | 17 | 17 | Yes | No | Yes | No | No | No | Largest one on back; multiple EMs | NA | NA | NA | No |
| LDB30 | Endemic Control | 2017 | EH | 30 | Male | White | NA | NA | NA | NA | NA | NA | NA | NA | NA | NA | NA | NA | NA | No |
| LDB31 | Endemic Control | 2017 | EH | 20 | Female | Hispanic or Latino | NA | NA | NA | NA | NA | NA | NA | NA | NA | NA | NA | NA | NA | No |
| LDB32 | EM+/STT- | 2017 | WI | 60 | Male | White | Yes, 9 | 14 | 17 | Yes | Yes | No | Yes | No | Yes | Upper left thigh | 10 | NA | NA | No |
| LDB33 | Endemic Control | 2017 | EH | 86 | Female | White | NA | NA | NA | NA | NA | NA | NA | NA | NA | NA | NA | NA | NA | No |
| LDB34 | EM+/STT- | 2017 | WI | 53 | Male | White | Yes, 6 | 8 | 4 | No | Yes | No | Yes | No | Yes | Left armpit | 4 | 1 | Doxycycline | No |
| LDB35 | Endemic Control | 2017 | WI | 61 | Male | White | NA | NA | NA | NA | NA | NA | NA | NA | NA | NA | NA | NA | NA | No |
| LDB36 | Endemic Control | 2017 | WI | 47 | Female | White | NA | NA | NA | NA | NA | NA | NA | NA | NA | NA | NA | NA | NA | No |
| LDB37 | STT+ | 2017 | WI | 68 | Female | White | No | 6 | 4 | Yes | No | Yes | Yes | No | Yes | 100+ spots, arms, legs, butt, chest, feet, stomach, disseminated disease | 7 | 1 | Doxycycline | No |
| LDB38 | Endemic Control | 2017 | EH | 59 | Female | White | NA | NA | NA | NA | NA | NA | NA | NA | NA | NA | NA | NA | NA | No |
| LDB39 | EM+/STT- | 2017 | EH | 57 | Male | White | Yes, 22 | 10 | 7 | Yes | Yes | No | Yes | No | Yes | Right upper arm | 7 | NA | NA | Yes |
| LDB40 | EM+/STT- | 2017 | EH | 58 | Male | White | No | 11 | 9 | Yes | Yes | No | No | Yes | Yes | Right thigh | 4 | NA | NA | No |
| LDB41 | EM+/STT- | 2017 | EH | 25 | Male | White | Yes, 2 | 14 | 14 | Yes | Yes | No | Yes | No | Yes | Right post knee | 2 | NA | NA | Yes |
| LDB42 | EM+/STT- | 2017 | WI | 70 | Female | White | Yes, 5 | 15 | 11 | Yes | No | Yes | No | Yes | Yes | Stomach and side | 4 | NA | NA | No |
| LDB43 | EM+/STT- | 2017 | WI | 17 | Male | White | Yes, 29 | 8 | 2 | No | Yes | No | No | Yes | No | Right hip | 1 | NA | NA | No |
| LDB44 | EM+/STT- | 2017 | WI | 62 | Male | White | Yes, 10 | 7 | 6 | Yes | Yes | No | No | Yes | Yes | upper arm | 9 | NA | NA | Yes |
| LDB45 | EM+/STT- | 2017 | WI | 54 | Female | White | No | 18 | 15 | Yes | Yes | No | No | Yes | Yes | Upper back | 3 | 2 | Cephalexin | No |
| LDB46 | EM+/STT- | 2017 | WI | 62 | Female | White | Yes, 12 | 7 | 7 | Yes | Yes | No | Yes | No | Yes | Inside right thigh | 11 | 1 | Doxycycline | No |
| LDB47 | Endemic Control | 2017 | EH | 69 | Male | White | NA | NA | NA | NA | NA | NA | NA | NA | NA | NA | NA | NA | NA | No |
| LDB48 | EM+/STT- | 2017 | EH | 30 | Male | White | Yes, 21 | 8 | 5 | Yes | Yes | No | Yes | No | Yes | Right upper arm | 14 | NA | NA | Yes |
| LDB49 | Endemic Control | 2017 | EH | 55 | Female | White | NA | NA | NA | NA | NA | NA | NA | NA | NA | NA | NA | NA | NA | No |
| LDB50 | EM+/STT- | 2017 | EH | 30 | Male | Hispanic or Latino | Yes, 13 | 8 | 5 | Yes | Yes | No | Yes | No | Yes | upper back | 10 | NA | NA | No |
| LDB51 | EM+/STT- | 2017 | EH | 25 | Male | Hispanic or Latino | Yes, 3 | 10 | 4 | Yes | Yes | No | No | Yes | Yes | Left hip | 3 | 1 | Doxycycline | No |
| LDB52 | EM+/STT- | 2017 | EH | 47 | Female | Hispanic or Latino | Yes, 12 | 11 | 4 | Yes | Yes | No | Yes | No | Yes | Right upper flank | 11 | NA | NA | No |
| LDB53 | Endemic Control | 2017 | EH | 28 | Female | White | NA | NA | NA | NA | NA | NA | NA | NA | NA | NA | NA | NA | NA | No |
| LDB54 | EM+/STT- | 2017 | EH | 64 | Female | White | Yes, 10 | 5 | 6 | No | No | Yes | No | No | Yes | right thigh, right forearm | unknown | NA | NA | No |
| LDB55 | EM+/STT- | 2017 | WI | 63 | Male | White | No | 6 | 4 | Yes | Yes | No | No | Yes | Yes | Right upper arm and back | 5 | NA | NA | No |
| LDB56 | EM+/STT- | 2017 | WI | 55 | Female | White | Yes, 25 | 16 | 16 | Yes | Yes | No | Yes | No | Yes | Left inner thigh | 20 | 1 | Doxycycline | No |
| LDB57 | EM+/STT- | 2017 | WI | 65 | Male | White | No | 11 | 10 | Yes | Yes | No | Yes | No | Yes | Upper right thigh | 3 | 1 | Doxycycline | No |
| LDB58 | EM+/STT- | 2017 | EH | 24 | Female | Hispanic or Latino | Yes, 9 | 10 | 6 | Yes | Yes | No | Yes | No | Yes | Right groin | 3 | NA | NA | No |
| LDB59 | Endemic Control | 2017 | WI | 66 | Male | White | NA | NA | NA | NA | NA | NA | NA | NA | NA | NA | NA | NA | NA | No |
| LDB60 | EM+/STT- | 2017 | EH | 44 | Male | Hispanic or Latino | Yes, 13 | 5 | 6 | Yes | Yes | No | No | Yes | Yes | Left lower leg | 7 | 1 | Amoxicillin | No |
| LDB61 | Endemic Control | 2017 | WI | 84 | Female | White | NA | NA | NA | NA | NA | NA | NA | NA | NA | NA | NA | NA | NA | No |
| LDB62 | EM+/STT- | 2017 | EH | 52 | Female | Hispanic or Latino | Yes, 14 | 7 | 9 | Yes | Yes | No | Yes | No | Yes | Right ankle | 7 | NA | NA | No |
| LDB63 | EM+/STT- | 2017 | EH | 53 | Male | White | No | 14 | 13 | Yes | Yes | No | Yes | No | Yes | Right shoulder | 14 | NA | NA | No |
| LDB64 | Endemic Control | 2017 | EH | 42 | Female | White | NA | NA | NA | NA | NA | NA | NA | NA | NA | NA | NA | NA | NA | No |
| LDB65 | EM+/STT- | 2017 | EH | 84 | Male | White | No | 18 | 12 | Yes | Yes | No | No | Yes | Yes | Left groin | unknown | NA | NA | No |
| LDB66 | EM+/STT- | 2017 | EH | 30 | Male | White | Yes, 38 | 16 | 14 | Yes | Yes | No | No | Yes | Yes | Left thigh | 2 | NA | NA | Yes |
| LDB67 | Endemic Control | 2017 | WI | 37 | Female | White | NA | NA | NA | NA | NA | NA | NA | NA | NA | NA | NA | NA | NA | No |
| LDB68 | Endemic Control | 2017 | EH | 70 | Male | White | NA | NA | NA | NA | NA | NA | NA | NA | NA | NA | NA | NA | NA | No |
| LDB69 | Endemic Control | 2017 | WI | 56 | Female | White | NA | NA | NA | NA | NA | NA | NA | NA | NA | NA | NA | NA | NA | No |
| LDB70 | Endemic Control | 2017 | WI | 28 | Female | White | NA | NA | NA | NA | NA | NA | NA | NA | NA | NA | NA | NA | NA | No |
| LDB71 | Endemic Control | 2017 | WI | 35 | Female | White | NA | NA | NA | NA | NA | NA | NA | NA | NA | NA | NA | NA | NA | No |
| LDB72 | Endemic Control | 2017 | WI | 37 | Female | White | NA | NA | NA | NA | NA | NA | NA | NA | NA | NA | NA | NA | NA | No |
| LDB73 | Endemic Control | 2017 | WI | 28 | Female | White | NA | NA | NA | NA | NA | NA | NA | NA | NA | NA | NA | NA | NA | No |
| LDB74 | Endemic Control | 2017 | WI | 45 | Male | White | NA | NA | NA | NA | NA | NA | NA | NA | NA | NA | NA | NA | NA | No |
| LDB75 | Endemic Control | 2017 | WI | 42 | Female | White | NA | NA | NA | NA | NA | NA | NA | NA | NA | NA | NA | NA | NA | No |
| LDB76 | Endemic Control | 2017 | WI | 38 | Female | White | NA | NA | NA | NA | NA | NA | NA | NA | NA | NA | NA | NA | NA | No |
| LDB77 | Endemic Control | 2017 | WI | 63 | Female | White | NA | NA | NA | NA | NA | NA | NA | NA | NA | NA | NA | NA | NA | No |
| LDB78 | Endemic Control | 2017 | WI | 34 | Female | White | NA | NA | NA | NA | NA | NA | NA | NA | NA | NA | NA | NA | NA | No |
| LDB79 | Endemic Control | 2017 | WI | 40 | Female | White | NA | NA | NA | NA | NA | NA | NA | NA | NA | NA | NA | NA | NA | No |
| LDB80 | Endemic Control | 2017 | WI | 68 | Female | White | NA | NA | NA | NA | NA | NA | NA | NA | NA | NA | NA | NA | NA | No |
| LDB81 | EM+/STT- | 2018 | WI | 59 | Female | White | Yes, 11 | 13 | 9 | Yes | Yes | No | No | Yes | Yes | Right side torso | 2 | NA | NA | No |
| LDB82 | EM+/STT- | 2018 | EH | 44 | Female | White | Yes, 1 | 9 | 3 | Yes | Yes | No | Yes | No | No | Left thigh | 1 | NA | NA | No |
| LDB83 | Endemic Control | 2018 | EH | 55 | Female | White | NA | NA | NA | NA | NA | NA | NA | NA | NA | NA | NA | NA | NA | No |
| LDB84 | Endemic Control | 2018 | EH | 31 | Male | White | NA | NA | NA | NA | NA | NA | NA | NA | NA | NA | NA | NA | NA | No |
| LDB85 | EM+/STT- | 2018 | EH | 64 | Male | White | Yes, 6 | 6 | 7 | Yes | Yes | No | No | Yes | Yes | Right leg | 7 | NA | NA | No |
| LDB86 | EM+/STT- | 2018 | WI | 56 | Male | White | Yes, 23 | 9 | 5 | Yes | Yes | No | Yes | Yes | Yes | Left thigh | 2 | 1 | Doxycycline | No |
| LDB87 | EM+/STT- | 2018 | WI | 53 | Female | White | Yes, 16 | 9 | 5 | Yes | Yes | No | No | Yes | Yes | Right side of neck | 6 | 2 | Doxycycline | No |
| LDB88 | EM+/STT- | 2018 | WI | 75 | Male | White | Yes, 26 | 5 | 6 | Yes | Yes | No | No | Yes | Yes | Right bicep | 4 | NA | NA | No |
| LDB89 | EM+/STT- | 2018 | WI | 71 | Male | White | No | 15 | 10 | No | Yes | No | No | Yes | Yes | Right upper inner thigh | 10 | 1 | Doxycycline | No |
| LDB90 | EM+/STT- | 2018 | EH | 75 | Male | White | No | 12 | 9 | Yes | Yes | No | Yes | No | Yes | Left forearm | Unknown | NA | NA | Yes |
| LDB91 | EM+/STT- | 2018 | EH | 54 | Male | Hispanic or Latino | Yes, 2 | 6 | 6 | Yes | Yes | No | Yes | No | Yes | Right upper thigh | 2 | NA | NA | Yes |
| LDB92 | EM+/STT- | 2018 | EH | 46 | Male | White | Yes, 13 | 12 | 9 | Yes | Yes | No | Yes | No | Yes | Right leg | 4 | 7 | Clinda | No |
| LDB93 | EM+/STT- | 2018 | EH | 56 | Male | White | No | 13 | 7 | Yes | Yes | No | Yes | No | Yes | Left hip | 3 | NA | NA | Yes |
| LDB94 | EM+/STT- | 2018 | EH | 43 | Male | Hispanic or Latino | Yes, 3 | 7 | 5 | Yes | Yes | No | No | Yes | Yes | Right buttocks | 4 | NA | NA | No |
| LDB95 | Endemic Control | 2018 | WI | 57 | Female | White | NA | NA | NA | NA | NA | NA | NA | NA | NA | NA | NA | NA | NA | No |
| LDB96 | EM+/STT- | 2018 | EH | 47 | Female | White | Yes, 2 | 7 | 4 | Yes | No | Yes | Yes | No | Yes | Right inguinal, Right buttock | 2 | NA | NA | Yes |
| LDB97 | EM+/STT- | 2018 | EH | 60 | Female | White | No | 9 | 8 | Yes | Yes | No | Yes | No | Yes | Left shin | 7 | NA | NA | Yes |
| LDB98 | EM+/STT- | 2018 | EH | 28 | Female | White | Yes, 15 | 14 | 12 | No | No | Yes | Yes | No | Yes | Right arm and Right leg | 2 | NA | NA | No |
| LDB99 | EM+/STT- | 2018 | WI | 34 | Female | White | No | 8 | 4 | No | Yes | No | No | Yes | No | Back of left knee | 1 | NA | NA | No |
| LDB100 | Endemic Control | 2018 | WI | 28 | Female | White | NA | NA | NA | NA | NA | NA | NA | NA | NA | NA | NA | NA | NA | No |
| LDB101 | Endemic Control | 2018 | EH | 65 | Male | White | NA | NA | NA | NA | NA | NA | NA | NA | NA | NA | NA | NA | NA | No |
| LDB102 | EM+/STT- | 2018 | EH | 88 | Male | White | No | 11 | 11 | Yes | Yes | No | Yes | No | No | Left groin | 1 | NA | NA | No |
| LDB103 | EM+/STT- | 2018 | EH | 40 | Female | White | No | 9 | 8 | Yes | Yes | No | Yes | No | Yes | Right arm | 3 | NA | NA | No |
| LDB104 | EM+/STT- | 2018 | EH | 23 | Male | Hispanic or Latino | Yes, 21 | 6 | 4 | No | Yes | No | No | Yes | Yes | Left ankle | 5 | NA | NA | No |
| LDB105 | Endemic Control | 2018 | WI | 37 | Female | White | NA | NA | NA | NA | NA | NA | NA | NA | NA | NA | NA | NA | NA | No |
| LDB106 | Endemic Control | 2018 | WI | 47 | Female | Asian | NA | NA | NA | NA | NA | NA | NA | NA | NA | NA | NA | NA | NA | No |
| LDB107 | Endemic Control | 2018 | EH | 26 | Male | Asian | NA | NA | NA | NA | NA | NA | NA | NA | NA | NA | NA | NA | NA | No |
| LDB108 | EM+/STT- | 2018 | EH | 55 | Female | Hispanic or Latino | No | 11 | 11 | Yes | Yes | No | No | No | Yes | Left upper thigh | 4 | NA | NA | No |
| LDB109 | Endemic Control | 2018 | EH | 28 | Male | Hispanic or Latino | NA | NA | NA | NA | NA | NA | NA | NA | NA | NA | NA | NA | NA | No |
| LDB110 | Endemic Control | 2018 | WI | 34 | Female | White | NA | NA | NA | NA | NA | NA | NA | NA | NA | NA | NA | NA | NA | No |
| LDB111 | Endemic Control | 2018 | WI | 31 | Female | White | NA | NA | NA | NA | NA | NA | NA | NA | NA | NA | NA | NA | NA | No |
| LDB112 | Endemic Control | 2019 | EH | 54 | Female | White | NA | NA | NA | NA | NA | NA | NA | NA | NA | NA | NA | NA | NA | No |
| LDB113 | EM+/STT- | 2019 | EH | 70 | Male | White | Yes, 4 | 7 | 5 | Yes | No | Yes | No | Yes | Yes | Both legs, back | 4 | 1 | Amoxicillin | Yes |
| LDB114 | EM+/STT- | 2019 | EH | 55 | Female | White | Yes, 7 | 8 | 5 | No | Yes | No | Yes | No | Yes | Left back | 1 | NA | NA | No |
| LDB115 | Endemic Control | 2019 | EH | 16 | Female | White | NA | NA | NA | NA | NA | NA | NA | NA | NA | NA | NA | NA | NA | No |
| LDB116 | EM+/STT- | 2019 | EH | 55 | Female | White | Yes, 14 | 13 | 7 | Yes | Yes | No | No | Yes | Yes | Left flank | 2 | NA | NA | Yes |
| LDB117 | EM+/STT- | 2019 | EH | 78 | Male | White | No | 8 | 12 | Yes | Yes | No | Yes | No | Yes | Left Thigh | 2 | 2 | Cephalexin | Yes |
| LDB118 | EM+/STT- | 2019 | WI | 22 | Male | White | Yes, 21 | 12 | 4 | Yes | Yes | No | Yes | No | Yes | Left inner thigh | 6 | 1 | Doxycycline | Yes |
| LDB119 | EM+/STT- | 2019 | WI | 63 | Male | White | Yes, 10 | 9 | 3 | Yes | Yes | No | No | Yes | Yes | Inner left thigh | 10 | NA | NA | Yes |
| LDB120 | Endemic Control | 2019 | WI | 62 | Female | White | NA | NA | NA | NA | NA | NA | NA | NA | NA | NA | NA | NA | NA | No |
| LDB121 | Endemic Control | 2019 | WI | 48 | Female | White | NA | NA | NA | NA | NA | NA | NA | NA | NA | NA | NA | NA | NA | No |
| LDB122 | STT+ | 2020 | EH | 62 | Male | White | No | 14 | 12 | Yes | Yes | No | Yes | No | Yes | Left arm | 5 | NA | NA | Yes |
| LDB123 | EM+/STT- | 2020 | EH | 28 | Male | Hispanic or Latino | Yes, 2 | 6 | 5 | Yes | Yes | No | Yes | No | Yes | Arm | 1 | NA | NA | No |
| LDB124 | EM+/STT- | 2020 | EH | 32 | Male | White | No | 8 | 7 | Yes | Yes | No | Yes | No | Yes | Right upper arm | 7 | NA | NA | No |
| LDB125 | STT+ | 2021 | WI | 42 | Male | White | No | 7 | 11 | Yes | Yes | No | No | No | Yes | Left inner thigh | 10 | 2 | Doxycycline | No |
| LDB126 | STT+ | 2021 | WI | 57 | Male | White | Yes, 17 | 7 | 5 | No | No | Yes | No | Yes | Yes | Face, legs, back, flanks, torso | 3 | 2 | Doxycycline | No |
| LDB127 | STT+ | 2021 | WI | 60 | Female | White | No | 7 | 5 | No | No | Yes | No | Yes | Yes | Posterior of right knee, upper right leg, back | Unknown | NA | NA | No |
| LDB128 | STT+ | 2021 | WI | 61 | Female | White | No | 7 | 5 | Yes | No | Yes | No | Yes | Yes | Entire body | 15 | 1 | Doxycycline | No |
| LDB129 | Endemic Control | 2021 | WI | 64 | Female | White | NA | NA | NA | NA | NA | NA | NA | NA | NA | NA | NA | NA | NA | No |
| LDB130 | EM+/STT- | 2021 | EH | 53 | Male | White | Yes, 13 | 9 | 7 | Yes | Yes | No | Yes | No | Yes | Right thigh | 3 | NA | NA | No |
| LDB131 | STT+ | 2021 | EH | 91 | Female | White | No | NA | NA | NA | NA | NA | NA | NA | NA | NA | NA | NA | NA | Yes |
| LDB132 | EM+/STT- | 2021 | EH | 61 | Male | White | Yes, 10 | 10 | 15 | Yes | Yes | No | Yes | No | Yes | Right thigh | 3 | NA | NA | No |
| LDB133 | EM+/STT- | 2021 | EH | 89 | Female | Hispanic or Latino | Yes, 7 | 6 | 8 | Yes | Yes | No | No | Yes | Yes | Right thigh | 7 | NA | NA | Yes |
| LDB134 | EM+/STT- | 2021 | EH | 51 | Male | White | Yes, 7 | 9 | 4 | No | Yes | No | Yes | No | Yes | Left flank | 7 | NA | NA | No |
| LDB135 | EM+/STT- | 2021 | EH | 31 | Male | Hispanic or Latino | Yes, 14 | 18 | 20 | Yes | Yes | No | Yes | No | Yes | Left flank | 14 | NA | NA | No |
| LDB136 | EM+/STT- | 2021 | EH | 25 | Male | White | No | 12 | 5 | No | Yes | No | No | Yes | Yes | Left Thigh | 3 | NA | NA | No |
| LDB137 | STT+ | 2021 | WI | 62 | Male | White | No | 15 | 13 | Yes | Yes | No | No | Yes | Yes | Upper back | 37 | 1 | Doxycycline | No |
| LDB138 | Endemic Control | 2021 | WI | 53 | Female | White | NA | NA | NA | NA | NA | NA | NA | NA | NA | NA | NA | NA | NA | No |
| LDB139 | Endemic Control | 2021 | WI | 49 | Male | White | NA | NA | NA | NA | NA | NA | NA | NA | NA | NA | NA | NA | NA | No |
| LDB140 | Endemic Control | 2021 | WI | 77 | Male | White | NA | NA | NA | NA | NA | NA | NA | NA | NA | NA | NA | NA | NA | No |
| LDB141 | Endemic Control | 2021 | WI | 63 | Female | White | NA | NA | NA | NA | NA | NA | NA | NA | NA | NA | NA | NA | NA | No |
| LDB142 | STT+ | 2021 | EH | 63 | Male | White | Yes, 42 | 10 | 7 | No | Yes | No | Yes | No | Yes | Armpit | 21 | NA | NA | Yes |
| LDB143 | Endemic Control | 2021 | WI | 64 | Female | White | NA | NA | NA | NA | NA | NA | NA | NA | NA | NA | NA | NA | NA | No |
| LDB144 | EM+/STT- | 2022 | EH | 56 | Female | Hispanic or Latino | Yes, 2 | 10 | 7 | No | Yes | No | Yes | No | Yes | Right abdomen | 3 | NA | NA | No |
| LDB145 | Endemic Control | 2021 | EH | 29 | Male | White | NA | NA | NA | NA | NA | NA | NA | NA | NA | NA | NA | NA | NA | No |
| LDB146 | Endemic Control | 2021 | EH | 25 | Female | Hispanic or Latino | NA | NA | NA | NA | NA | NA | NA | NA | NA | NA | NA | NA | NA | No |
| LDB147 | STT+ | 2022 | WI | 59 | Male | White | No | 13 | 8 | No | No | Yes | No | Yes | Yes | Chest, back, arms, legs | 4 | NA | NA | No |
| LDB148 | Endemic Control | 2022 | WI | 62 | Male | White | NA | NA | NA | NA | NA | NA | NA | NA | NA | NA | NA | NA | NA | No |
| LDB149 | Endemic Control | 2022 | WI | 49 | Male | White | NA | NA | NA | NA | NA | NA | NA | NA | NA | NA | NA | NA | NA | No |
| LDB150 | Endemic Control | 2022 | WI | 24 | Female | White | NA | NA | NA | NA | NA | NA | NA | NA | NA | NA | NA | NA | NA | No |
| LDB151 | EM+/STT- | 2022 | EH | 47 | Female | Hispanic or Latino | No | 13 | 7 | No | Yes | No | Yes | No | Yes | Right lower leg | Unknown | NA | NA | Yes |
| LDB152 | Endemic Control | 2022 | EH | 27 | Female | White | NA | NA | NA | NA | NA | NA | NA | NA | NA | NA | NA | NA | NA | No |
| LDB153 | Endemic Control | 2022 | EH | 21 | Female | Hispanic or Latino | NA | NA | NA | NA | NA | NA | NA | NA | NA | NA | NA | NA | NA | No |
| LDB154 | Endemic Control | 2022 | EH | 22 | Female | White | NA | NA | NA | NA | NA | NA | NA | NA | NA | NA | NA | NA | NA | No |
| LDB155 | Endemic Control | 2022 | WI | 25 | Female | White | NA | NA | NA | NA | NA | NA | NA | NA | NA | NA | NA | NA | NA | No |
| LDB156 | EM+/STT- | 2022 | EH | 23 | Male | White | Yes, 2 | 13 | 10 | Yes | Yes | No | Yes | No | Yes | Left back of knee | 1 | 1 | Doxycycline | No |
| LDB157 | EM+/STT- | 2022 | EH | 55 | Female | Hispanic or Latino | No | 7 | 6 | Yes | Yes | No | No | Yes | Yes | Right leg | 4 | NA | NA | No |
| LDB158 | STT+ | 2022 | EH | 69 | Male | White | No | 15 | 16 | No | Yes | No | Yes | No | No | Right flank | 1 | NA | NA | No |
| LDB159 | EM+/STT- | 2022 | EH | 52 | Male | Hispanic or Latino | Yes, 7 | 8 | 3 | Yes | Yes | No | No | Yes | Yes | Left hip | 5 | NA | NA | No |
| LDB160 | EM+/STT- | 2022 | EH | 60 | Male | Hispanic or Latino | No | 20 | 15 | Yes | Yes | No | Yes | No | Yes | Left back | 21 | NA | NA | No |
| LDB161 | Endemic Control | 2022 | EH | 66 | Female | White | NA | NA | NA | NA | NA | NA | NA | NA | NA | NA | NA | NA | NA | No |
| LDB162 | STT+ | 2022 | WI | 21 | Male | White | Yes, N/A | 14 | 13 | Yes | No | Yes | Yes | No | Yes | Upper arm, back, chest, legs | 7 | 1 | Doxycycline | No |
| LDB163 | STT+ | 2022 | WI | 62 | Female | White | No | 7 | 5 | Yes | No | Yes | No | Yes | Yes | Abdomen and legs | 17 | 2 | Doxycycline | No |
| LDB164 | EM+/STT- | 2022 | EH | 46 | Male | White | No | 11 | 6 | Yes | Yes | No | Yes | No | Yes | Right post knee | 5 | NA | NA | No |
| LDB165 | Endemic Control | 2022 | EH | 28 | Female | Hispanic or Latino | NA | NA | NA | NA | NA | NA | NA | NA | NA | NA | NA | NA | NA | No |
| LDB166 | Endemic Control | 2022 | EH | 32 | Female | Hispanic or Latino | NA | NA | NA | NA | NA | NA | NA | NA | NA | NA | NA | NA | NA | No |
| LDB167 | Endemic Control | 2022 | EH | 63 | Male | White | NA | NA | NA | NA | NA | NA | NA | NA | NA | NA | NA | NA | NA | No |
| LDB168 | Endemic Control | 2022 | EH | 54 | Male | White | NA | NA | NA | NA | NA | NA | NA | NA | NA | NA | NA | NA | NA | No |
| LDB169 | EM+/STT- | 2022 | EH | 59 | Male | White | No | 9 | 4 | No | Yes | No | No | Yes | Yes | Right back | 4 | NA | NA | Yes |
| LDB170 | EM+/STT- | 2022 | EH | 26 | Male | White | Yes, 4 | 8 | 5 | Yes | Yes | No | No | Yes | Yes | Left lower back | 2 | NA | NA | No |
| LDB171 | STT+ | 2022 | WI | 38 | Male | White | No | 8 | 4 | Yes | No | Yes | No | Yes | Yes | chest, arms and back | 8 | 1 | Doxycycline | No |
| LDB172 | Endemic Control | 2022 | WI | 24 | Female | White | NA | NA | NA | NA | NA | NA | NA | NA | NA | NA | NA | NA | NA | No |
| LDB173 | Endemic Control | 2022 | WI | 56 | Female | White | NA | NA | NA | NA | NA | NA | NA | NA | NA | NA | NA | NA | NA | No |
| LDB174 | Endemic Control | 2022 | WI | 59 | Female | White | NA | NA | NA | NA | NA | NA | NA | NA | NA | NA | NA | NA | NA | No |
| LDB175 | Endemic Control | 2022 | WI | 41 | Female | White | NA | NA | NA | NA | NA | NA | NA | NA | NA | NA | NA | NA | NA | No |
| LDB176 | Endemic Control | 2022 | WI | 61 | Male | White | NA | NA | NA | NA | NA | NA | NA | NA | NA | NA | NA | NA | NA | No |
| LDB177 | Endemic Control | 2022 | WI | 22 | Female | White | NA | NA | NA | NA | NA | NA | NA | NA | NA | NA | NA | NA | NA | No |
| LDB178 | Endemic Control | 2023 | EH | 26 | Female | Hispanic or Latino | NA | NA | NA | NA | NA | NA | NA | NA | NA | NA | NA | NA | NA | No |
| LDB179 | Endemic Control | 2023 | EH | 29 | Female | Hispanic or Latino | NA | NA | NA | NA | NA | NA | NA | NA | NA | NA | NA | NA | NA | No |
| LDB180 | Endemic Control | 2023 | EH | 46 | Female | White | NA | NA | NA | NA | NA | NA | NA | NA | NA | NA | NA | NA | NA | No |
| LDB181 | EM+/STT- | 2023 | EH | 75 | Female | Hispanic or Latino | Yes, 4 | 3 | 6 | No | Yes | No | No | Yes | Yes | Left calf | 3 | NA | NA | No |
| LDB182 | Endemic Control | 2023 | EH | 19 | Male | White | NA | NA | NA | NA | NA | NA | NA | NA | NA | NA | NA | NA | NA | No |
| LDB183 | EM+/STT- | 2023 | EH | 62 | Female | White | No | 14 | 4 | Yes | Yes | No | Yes | No | Yes | Left upper abdomen | 1 | NA | NA | Yes |
| LDB184 | STT+ | 2023 | EH | 48 | Male | Hispanic or Latino | No | 4 | 4 | Yes | Yes | No | Yes | No | Yes | Right Thigh | 14 | NA | NA | Yes |
| LDB185 | EM+/STT- | 2023 | EH | 55 | Female | White | No | 15 | 8 | Yes | Yes | No | Yes | No | Yes | Left axila | 7 | NA | NA | No |
| LDB186 | Endemic Control | 2023 | WI | 42 | Female | White | NA | NA | NA | NA | NA | NA | NA | NA | NA | NA | NA | NA | NA | No |
| LDB187 | EM+/STT- | 2023 | EH | 53 | Male | White | No | 11 | 6 | Yes | Yes | No | No | Yes | Yes | Left flank | 1 | NA | NA | No |
| LDB188 | EM+/STT- | 2023 | EH | 38 | Female | Hispanic or Latino | No | 19 | 13 | Yes | Yes | No | Yes | No | Yes | Left anterior shoulder | 6 | NA | NA | No |
| LDB189 | EM+/STT- | 2023 | EH | 46 | Male | Hispanic or Latino | Yes, 13 | 12 | 8 | Yes | Yes | No | Yes | No | Yes | Back | 15 | NA | NA | No |
| LDB190 | EM+/STT- | 2023 | EH | 31 | Female | White | No | 7 | 6 | Yes | Yes | No | Yes | No | Yes | Left lower back | 2 | NA | NA | Yes |
| LDB191 | Endemic Control | 2023 | WI | 40 | Male | White | NA | NA | NA | NA | NA | NA | NA | NA | NA | NA | NA | NA | NA | No |
| LDB192 | Endemic Control | 2023 | WI | 77 | Male | White | NA | NA | NA | NA | NA | NA | NA | NA | NA | NA | NA | NA | NA | No |
| LDB193 | Endemic Control | 2023 | WI | 57 | Female | White | NA | NA | NA | NA | NA | NA | NA | NA | NA | NA | NA | NA | NA | No |
| LDB194 | Endemic Control | 2023 | WI | 57 | Female | White | NA | NA | NA | NA | NA | NA | NA | NA | NA | NA | NA | NA | NA | No |
| LDB195 | Endemic Control | 2023 | WI | 60 | Female | White | NA | NA | NA | NA | NA | NA | NA | NA | NA | NA | NA | NA | NA | No |

Supplemental Table 3. Demographic Data for blinded panel from the Lyme Disease Biobank (LDB) comprised of 100 endemic controls (E C), 79 clinically positive but standard two-tier test negative (EM+/STT-), and 16 standard two-tier test positive (STT+) samples.
