# Supplementary material for: The multiplexed single-tier InBios Lyme Detect Multiplex ELISA is more sensitive than standard two-tier tests in the early stages of Lyme disease
Source: J Clin Microbiol. 2025 Oct 9;63(11):e00629-25. doi: 10.1128/jcm.00629-25 (PMC12607815; doi:10.1128/jcm.00629-25)
Supplement: Table S4 — Demographic data for 66 unblinded lookalike condition samples including 15 lupus (LGC and Sanguine Biosciences), 14 multiple sclerosis (LGC and Sanguine Biosciences), 15 fibromyalgia (Sanguine Biosciences), 10 rheumatoid arthritis (LGC), and 12 syphilis (Precision for Medicine) samples. [file jcm.00629-25-s0004.docx]

|  | | | | | | | Does patient report or recall tick bite? | Size of Rash | | Rash characteristics | | | | | | | Days you had rash | Antibiotic Duration (prophylactic) | Name of antibiotic (prophylactic) | Past history of Lyme disease |
| --- | --- | --- | --- | --- | --- | --- | --- | --- | --- | --- | --- | --- | --- | --- | --- | --- | --- | --- | --- | --- |
| Study Label | *Classification* | Season | Site | Age | Gender | Race | Response/ days since tick bite | Length (cm) | Width (cm) | History of Expansion | Single EM | Multiple EM | Classic "Bulls-Eye" | Homogenous Erythema | Rash prior to today: | Location(s) (please describe) | Open-Ended Response | Duration (days) | Response | Response |
| Lu1 | Lupus | NA | NA | 29 | Male | Female | NA | NA | NA | NA | NA | NA | NA | NA | NA | NA | NA | NA | NA | NA |
| Lu2 | Lupus | NA | NA | 28 | Female | Caucasian | NA | NA | NA | NA | NA | NA | NA | NA | NA | NA | NA | NA | NA | NA |
| Lu3 | Lupus | NA | NA | 39 | Female | Caucasian | NA | NA | NA | NA | NA | NA | NA | NA | NA | NA | NA | NA | NA | NA |
| Lu4 | Lupus | NA | NA | 40 | Female | Asian | NA | NA | NA | NA | NA | NA | NA | NA | NA | NA | NA | NA | NA | NA |
| Lu5 | Lupus | NA | NA | 44 | Female | Caucasian | NA | NA | NA | NA | NA | NA | NA | NA | NA | NA | NA | NA | NA | NA |
| Lu6 | Lupus | NA | NA | 47 | Female | Caucasian | NA | NA | NA | NA | NA | NA | NA | NA | NA | NA | NA | NA | NA | NA |
| Lu7 | Lupus | NA | NA | 80 | Male | Caucasian | NA | NA | NA | NA | NA | NA | NA | NA | NA | NA | NA | NA | NA | NA |
| Lu8 | Lupus | NA | NA | 55 | Female | Caucasian | NA | NA | NA | NA | NA | NA | NA | NA | NA | NA | NA | NA | NA | NA |
| Lu9 | Lupus | NA | NA | 36 | Female | Caucasian | NA | NA | NA | NA | NA | NA | NA | NA | NA | NA | NA | NA | NA | NA |
| Lu10 | Lupus | NA | NA | 44 | Female | Asian | NA | NA | NA | NA | NA | NA | NA | NA | NA | NA | NA | NA | NA | NA |
| Lu11 | Lupus | NA | NA | NA | NA | NA | NA | NA | NA | NA | NA | NA | NA | NA | NA | NA | NA | NA | NA | NA |
| Lu12 | Lupus | NA | NA | NA | NA | NA | NA | NA | NA | NA | NA | NA | NA | NA | NA | NA | NA | NA | NA | NA |
| Lu13 | Lupus | NA | NA | NA | NA | NA | NA | NA | NA | NA | NA | NA | NA | NA | NA | NA | NA | NA | NA | NA |
| Lu14 | Lupus | NA | NA | NA | NA | NA | NA | NA | NA | NA | NA | NA | NA | NA | NA | NA | NA | NA | NA | NA |
| Lu15 | Lupus | NA | NA | NA | NA | NA | NA | NA | NA | NA | NA | NA | NA | NA | NA | NA | NA | NA | NA | NA |
| MS1 | Multiple Sclerosis | NA | NA | 37 | Female | Caucasian | NA | NA | NA | NA | NA | NA | NA | NA | NA | NA | NA | NA | NA | NA |
| MS2 | Multiple Sclerosis | NA | NA | 49 | Female | Caucasian | NA | NA | NA | NA | NA | NA | NA | NA | NA | NA | NA | NA | NA | NA |
| MS3 | Multiple Sclerosis | NA | NA | 70 | Female | Caucasian | NA | NA | NA | NA | NA | NA | NA | NA | NA | NA | NA | NA | NA | NA |
| MS4 | Multiple Sclerosis | NA | NA | 42 | Female | Caucasian | NA | NA | NA | NA | NA | NA | NA | NA | NA | NA | NA | NA | NA | NA |
| MS5 | Multiple Sclerosis | NA | NA | 52 | Male | Caucasian | NA | NA | NA | NA | NA | NA | NA | NA | NA | NA | NA | NA | NA | NA |
| MS6 | Multiple Sclerosis | NA | NA | 21 | Female | Caucasian | NA | NA | NA | NA | NA | NA | NA | NA | NA | NA | NA | NA | NA | NA |
| MS7 | Multiple Sclerosis | NA | NA | 60 | Female | Caucasian | NA | NA | NA | NA | NA | NA | NA | NA | NA | NA | NA | NA | NA | NA |
| MS8 | Multiple Sclerosis | NA | NA | 55 | Female | Caucasian | NA | NA | NA | NA | NA | NA | NA | NA | NA | NA | NA | NA | NA | NA |
| MS9 | Multiple Sclerosis | NA | NA | 77 | Male | Caucasian | NA | NA | NA | NA | NA | NA | NA | NA | NA | NA | NA | NA | NA | NA |
| MS11 | Multiple Sclerosis | NA | NA | NA | NA | NA | NA | NA | NA | NA | NA | NA | NA | NA | NA | NA | NA | NA | NA | NA |
| MS12 | Multiple Sclerosis | NA | NA | NA | NA | NA | NA | NA | NA | NA | NA | NA | NA | NA | NA | NA | NA | NA | NA | NA |
| MS13 | Multiple Sclerosis | NA | NA | NA | NA | NA | NA | NA | NA | NA | NA | NA | NA | NA | NA | NA | NA | NA | NA | NA |
| MS14 | Multiple Sclerosis | NA | NA | NA | NA | NA | NA | NA | NA | NA | NA | NA | NA | NA | NA | NA | NA | NA | NA | NA |
| MS15 | Multiple Sclerosis | NA | NA | NA | NA | NA | NA | NA | NA | NA | NA | NA | NA | NA | NA | NA | NA | NA | NA | NA |
| Fi1 | Fibromyalgia | NA | NA | NA | NA | NA | NA | NA | NA | NA | NA | NA | NA | NA | NA | NA | NA | NA | NA | NA |
| Fi2 | Fibromyalgia | NA | NA | NA | NA | NA | NA | NA | NA | NA | NA | NA | NA | NA | NA | NA | NA | NA | NA | NA |
| Fi3 | Fibromyalgia | NA | NA | NA | NA | NA | NA | NA | NA | NA | NA | NA | NA | NA | NA | NA | NA | NA | NA | NA |
| Fi4 | Fibromyalgia | NA | NA | NA | NA | NA | NA | NA | NA | NA | NA | NA | NA | NA | NA | NA | NA | NA | NA | NA |
| Fi5 | Fibromyalgia | NA | NA | NA | NA | NA | NA | NA | NA | NA | NA | NA | NA | NA | NA | NA | NA | NA | NA | NA |
| Fi6 | Fibromyalgia | NA | NA | NA | NA | NA | NA | NA | NA | NA | NA | NA | NA | NA | NA | NA | NA | NA | NA | NA |
| Fi7 | Fibromyalgia | NA | NA | NA | NA | NA | NA | NA | NA | NA | NA | NA | NA | NA | NA | NA | NA | NA | NA | NA |
| Fi8 | Fibromyalgia | NA | NA | NA | NA | NA | NA | NA | NA | NA | NA | NA | NA | NA | NA | NA | NA | NA | NA | NA |
| Fi9 | Fibromyalgia | NA | NA | NA | NA | NA | NA | NA | NA | NA | NA | NA | NA | NA | NA | NA | NA | NA | NA | NA |
| Fi10 | Fibromyalgia | NA | NA | NA | NA | NA | NA | NA | NA | NA | NA | NA | NA | NA | NA | NA | NA | NA | NA | NA |
| Fi11 | Fibromyalgia | NA | NA | NA | NA | NA | NA | NA | NA | NA | NA | NA | NA | NA | NA | NA | NA | NA | NA | NA |
| Fi12 | Fibromyalgia | NA | NA | NA | NA | NA | NA | NA | NA | NA | NA | NA | NA | NA | NA | NA | NA | NA | NA | NA |
| Fi13 | Fibromyalgia | NA | NA | NA | NA | NA | NA | NA | NA | NA | NA | NA | NA | NA | NA | NA | NA | NA | NA | NA |
| Fi14 | Fibromyalgia | NA | NA | NA | NA | NA | NA | NA | NA | NA | NA | NA | NA | NA | NA | NA | NA | NA | NA | NA |
| Fi15 | Fibromyalgia | NA | NA | NA | NA | NA | NA | NA | NA | NA | NA | NA | NA | NA | NA | NA | NA | NA | NA | NA |
| RA1 | Rheumatoid Arthritis | NA | NA | 83 | Female | Caucasian | NA | NA | NA | NA | NA | NA | NA | NA | NA | NA | NA | NA | NA | NA |
| RA2 | Rheumatoid Arthritis | NA | NA | 41 | Male | Caucasian | NA | NA | NA | NA | NA | NA | NA | NA | NA | NA | NA | NA | NA | NA |
| RA3 | Rheumatoid Arthritis | NA | NA | 86 | Female | Caucasian | NA | NA | NA | NA | NA | NA | NA | NA | NA | NA | NA | NA | NA | NA |
| RA4 | Rheumatoid Arthritis | NA | NA | 88 | Female | Caucasian | NA | NA | NA | NA | NA | NA | NA | NA | NA | NA | NA | NA | NA | NA |
| RA5 | Rheumatoid Arthritis | NA | NA | 77 | Female | Caucasian | NA | NA | NA | NA | NA | NA | NA | NA | NA | NA | NA | NA | NA | NA |
| RA6 | Rheumatoid Arthritis | NA | NA | 86 | Female | Caucasian | NA | NA | NA | NA | NA | NA | NA | NA | NA | NA | NA | NA | NA | NA |
| RA7 | Rheumatoid Arthritis | NA | NA | 89 | Female | Caucasian | NA | NA | NA | NA | NA | NA | NA | NA | NA | NA | NA | NA | NA | NA |
| RA8 | Rheumatoid Arthritis | NA | NA | 83 | Female | Asian | NA | NA | NA | NA | NA | NA | NA | NA | NA | NA | NA | NA | NA | NA |
| RA9 | Rheumatoid Arthritis | NA | NA | 86 | Male | Caucasian | NA | NA | NA | NA | NA | NA | NA | NA | NA | NA | NA | NA | NA | NA |
| RA10 | Rheumatoid Arthritis | NA | NA | >89 | Female | Caucasian | NA | NA | NA | NA | NA | NA | NA | NA | NA | NA | NA | NA | NA | NA |
| Sy1 | Syphilis | 8/15/2012 | NA | 58 | Male | NA | NA | NA | NA | NA | NA | NA | NA | NA | NA | NA | NA | NA | NA | NA |
| Sy2 | Syphilis | 8/15/2012 | NA | 50 | Male | NA | NA | NA | NA | NA | NA | NA | NA | NA | NA | NA | NA | NA | NA | NA |
| Sy3 | Syphilis | 8/15/2012 | NA | 50 | Male | NA | NA | NA | NA | NA | NA | NA | NA | NA | NA | NA | NA | NA | NA | NA |
| Sy4 | Syphilis | 8/15/2012 | NA | 49 | Male | NA | NA | NA | NA | NA | NA | NA | NA | NA | NA | NA | NA | NA | NA | NA |
| Sy5 | Syphilis | 8/15/2012 | NA | 44 | Male | NA | NA | NA | NA | NA | NA | NA | NA | NA | NA | NA | NA | NA | NA | NA |
| Sy6 | Syphilis | 8/15/2012 | NA | 40 | Female | NA | NA | NA | NA | NA | NA | NA | NA | NA | NA | NA | NA | NA | NA | NA |
| Sy7 | Syphilis | 8/15/2012 | NA | 40 | Male | NA | NA | NA | NA | NA | NA | NA | NA | NA | NA | NA | NA | NA | NA | NA |
| Sy8 | Syphilis | 8/15/2012 | NA | 49 | Female | NA | NA | NA | NA | NA | NA | NA | NA | NA | NA | NA | NA | NA | NA | NA |
| Sy9 | Syphilis | 8/15/2012 | NA | 46 | Female | NA | NA | NA | NA | NA | NA | NA | NA | NA | NA | NA | NA | NA | NA | NA |
| Sy10 | Syphilis | 8/15/2012 | NA | 28 | Female | NA | NA | NA | NA | NA | NA | NA | NA | NA | NA | NA | NA | NA | NA | NA |
| Sy11 | Syphilis | 8/15/2012 | NA | 43 | Male | NA | NA | NA | NA | NA | NA | NA | NA | NA | NA | NA | NA | NA | NA | NA |
| Sy12 | Syphilis | 8/15/2012 | NA | 45 | Female | NA | NA | NA | NA | NA | NA | NA | NA | NA | NA | NA | NA | NA | NA | NA |

Supplemental Table 4. Demographic data for 66 unblinded lookalike condition samples including 15 Lupus (LGC and Sanguine Biosciences), 14 multiple sclerosis (LGC and Sanguine Biosciences), 15 fibromyalgia (Sanguine Biosciences), 10 rheumatoid arthritis (LGC), and 12 Syphilis (Precision for Medicine).
